# Toward a formal theory of proactivity

**DOI:** 10.3758/s13415-021-00884-y

**Published:** 2021-03-15

**Authors:** F. Lieder, G. Iwama

**Affiliations:** Rationality Enhancement Group, Max Planck Institute for Intelligent Systems, Tübingen, Max Planck Ring 4, D-72076 Tübingen, Germany

**Keywords:** Proactivity, Meta-control, Cognitive control, Proactive control, Computational modeling

## Abstract

Beyond merely reacting to their environment and impulses, people have the remarkable capacity to proactively set and pursue their own goals. The extent to which they leverage this capacity varies widely across people and situations. The goal of this article is to propose and evaluate a model of proactivity and reactivity. We proceed in three steps. First, we model proactivity in a widely used cognitive control task known as the AX Continuous Performance Task (AX-CPT). Our theory formalizes an important aspect of proactivity as meta-control over proactive and reactive control. Second, we perform a quantitative model comparison to identify the number and nature of meta-control decisions that are involved in the regulation of proactive behavior. Our findings suggest that individual differences in proactivity are governed by two independent meta-control decisions, namely deciding whether to set an intention for what to do in a future situation and deciding whether to recall one’s intentions when the situation occurs. Third, we test the assumptions and qualitative predictions of the winning model against data from numerous experiments varying the incentives, cognitive load, and statistical structure of the task. Our results suggest that proactivity can be understood in terms of computational models of meta-control. Future work will extend our models from proactive control in the AX-CPT to proactive goal creation and goal pursuit in the real world.

**Supplementary Information:**

The online version contains supplementary material available at 10.3758/s13415-021-00884-y.

## Introduction

Neuroscience and psychology have extensively studied how the brain processes and reacts to external stimuli and how those propensities are shaped by learning. This has led to significant progress in our understanding of important processes, such as habit formation, and the underlying neural mechanisms, such as reinforcement learning (Schultz et al., 1997). However, these theories do not address an essential element of what makes us human. That is, people do not merely react to their immediate environment and their drives and impulses. They also take initiative to set and pursue their own goals even if nothing in their immediate surroundings would suggest it. This quality is known as *proactivity* (Parker et al., 2010). So far, proactivity has been primarily studied in organizational studies, management sciences, and applied psychology, whereas part of its underlying cognitive mechanisms have been studied in the field of cognitive neuroscience (Braver, 2012). Proactivity is strongly associated with motivation and positive outcomes in real-world settings. For example, self-reported differences in proactivity in everyday life have been found to be predictors of job performance, career success, and career satisfaction (Judge & Kammeyer-Mueller, 2007). Not surprisingly, proactivity also is correlated with conscientiousness, self-efficacy, and responsibility for change (Tornau & Frese, 2013). Despite all the benefits of proactivity, high levels of proactivity are relatively rare (Seibert et al., 1999). Whether people think and act proactively depends on motivational, dispositional, and situational factors (Parker et al., 2019). In the absence of proactivity, human behavior is frequently controlled by stimulus-driven habitual or Pavlovian mechanisms instead of reflective and goal-directed decision mechanisms (Dolan & Dayan, 2013; van der van der Meer et al., 2012). We refer to this mode of behavioral control as *reactivity*.

By contrast, we define proactivity as the set of mechanisms that generate goal-directed behavior through the exertion of some form of cognitive control. This includes at least two distinct classes of mechanisms. The first pathway to proactive behavior is to set intentions in anticipation of future situations, actively maintain them in working memory, and then enact them when the anticipated situation occurs. For instance, if your fridge is empty, you may anticipate the need to override your habit to go home straight from work by the goal-directed behavior of stopping at the supermarket on your way home. Based on the anticipation that you will pass by the supermarket, you may start exerting cognitive control while you are still at work to create and actively memorize the implementation intention: “When the bus announces the stop next to the supermarket, then I will request it to stop and get out.” Recent research in cognitive neuroscience has begun to study this capacity under the heading of *proactive control* (Braver, 2012). Proactive control and its neural underpinnings have been studied in working memory paradigms (Braver, 2012; Burgess et al., 2011) and cognitive control tasks (Mäki-Marttunen et al., 2019a, 2019b). In working memory paradigms, proactive control manifests as the active maintenance of the intention to press a button if the test item matches one of the items to be memorized throughout the delay period. In cognitive control tasks, proactive control manifests as people initiating their response to an anticipated stimulus before it has even appeared based on the information provided by a predictive cue. A second pathway to proactive behavior comprises the stimulus-triggered recall of a goal or intention and the exertion of cognitive control to resolve conflicts between the recalled intention and default activities (automaticity). This mechanism is known as *reactive control* (Braver, 2012). For instance, if you are daydreaming on your way home, then the announcement of the next bus stop might reactivate your goal to go shopping. This, in turn, might prompt you to exert cognitive control to stop daydreaming, set the intention to stop the bus, and enact it immediately. Our definitions of proactivity and reactivity should not be confused with the notions of proactive control and reactive control as defined in the DMC framework. Rather, our notion of proactivity subsumes both proactive and reactive control and reactivity denotes the stimulus-driven automatic behavior that occurs in the absence of either form of cognitive control.

**Fig. 1:**
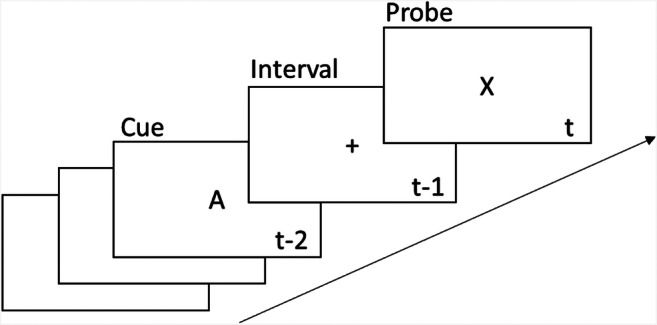
Illustration of the AX Continuous Performance Task

Proactive and reactive control have been extensively studied in a paradigm called the AX Continuous Performance Task (AX-CPT). As illustrated in Fig. 1, the AX-CPT presents participants with a stream of letters that are grouped into pairs. The first letter of each pair is called the *cue*, and the second letter is called the *probe*. There are two types of cues, called A cues and B cues, and two types of probes, called X probes and Y probes. The participant’s task is to detect AX trials, that is trials in which the pair comprises an A cue and an X probe by pressing button 1 and to press button 2 for all other pairs. Critically, in the standard AX-CPT, the frequencies of the four different trial types (AX, AY, BX, and BY) are such that an A is much more like to be followed by an X than by a Y, and there is a long delay between the cue and the probe. This allows participants to mentally prepare their response to probe even before it appears. For instance, when a participant sees an A cue, they might resolve to press button 1 as soon as the probe appears. This is an example of proactive control. On BX trials, by contrast, participants often have to engage in reactive control to override their habit to press button 1 when they see an X probe.

Proactivity has been found to be highly variable in laboratory paradigms (Braver, 2012) and in the real-world (Seibert et al., 1999). The Dual Mechanisms of Control framework (Braver, 2012) seeks to explain this variability in terms of differences in the extent to which people rely proactive control and reactive control. Despite initial modeling work (De Pisapia & Braver, 2006; Reynolds et al., 2006), the computational principles and algorithmic mechanisms of how people decide when to engage proactive control and when to engage reactive control remain unknown, and several theoretical conundrums remain to be resolved. For instance, it is still debated whether proactive control and reactive control are competing or complementary forms of control (Gonthier et al., 2016; Mäki-Marttunen et al., 2019a). Furthermore, it remains unclear how many and which meta-control decisions govern the variability in cognitive control within and across people. For instance, it is currently controversial whether people inhibit the intentions they have set in anticipation of one event when another event occurs that renders their intentions maladaptive (Mäki-Marttunen et al., 2018). The findings reviewed by Braver (2012) suggested that the frequency with which people engage proactive control increases with factors that make it more valuable (e.g., incentives) and decreases with factors that make it costlier (e.g., working memory load). These findings are congruent with the theory that people make rational use of their limited cognitive resources (Lieder & Griffiths, 2020) and the rational cost-benefit analysis postulated by the Expected Value of Control theory (Lieder et al., 2018; Shenhav et al., 2017). According to this theory, the identity and intensity of cognitive control signals are chosen to maximize the expected reward of performing the task minus the cost of control. The optimal control signal usually has an intermediate intensity, because stronger control signals are costlier. We hypothesize that the exertion of proactive and reactive control is governed by an equivalent rational cost-benefit analysis. Whether this hypothesis holds up to the scrutiny of testing its quantitative predictions remains to be seen. Last but not least, there is still a large gap between the low-level concepts of proactive and reactive control studied in cognitive neuroscience and the high-level concepts of proactivity and reactivity studied in organizational psychology and the management sciences.

We address these open theoretical questions by formalizing the foundational ideas of the Dual Mechanisms of Control framework (Braver, 2012) with a formal computational model of meta-control over proactive control and reactive control in the AX-CPT and testing its predictions against human performance in five previously conducted experiments. Our model builds on previous findings suggesting that the brain selects between alternative control mechanisms (*meta-control*) according to some kind of cost-benefit analysis (Boureau et al., 2015; Daw et al., 2005; Keramati et al., 2011; Lieder et al., 2018; Lieder & Griffiths, 2017; Shenhav et al., 2017). Viewing proactive control and reactive control as two complementary mechanisms of proactivity and goal-directed behavior, we developed and tested a formal computational model of the meta-control decisions that determine whether, when, and how a person engages proactive and/or reactive control, and how those meta-control decisions depend on situational and personal factors. The resulting meta-control model allowed us to explain individual differences in proactivity and how people’s propensities to engage proactive control and reactive control depend on incentives for speed and accuracy (Mäki-Marttunen et al., 2019b), cognitive load (Mäki-Marttunen et al., 2019b), the statistical structure of the task environment (Gonthier et al., 2016; Redick, 2014), and working memory capacity (Redick, 2014).

The outline of this paper is straightforward: we first introduce four alternative meta-control models of proactivity. We then test these models against each other and apply the best model to explain the findings of numerous experiments that investigated proactive control using the AX Continuous Performance Task (AX-CPT). We find that the available data is best explained by the assumption that people make two independent meta-control decisions about the engagement of proactive control and reactive control, respectively. Our findings suggest that individual differences in proactivity can be understood in terms of formal, rational models of how people tradeoff the costs and benefits of engaging in cognitive control. We close the paper by discussing directions for future work.

## Modeling the meta-control mechanisms of proactivity in the AX-CPT

The coexistence of proactive and reactive control in the AX-CP task makes it a good testbed for modeling proactive control, reactive control, and the meta-control processes that determine whether a person acts proactively or reactively. We therefore formulate our meta-control model of proactivity for the AX-CPT.

To understand the meta-control mechanisms in the AX-CPT, we developed a computational-level theory model of meta-control over proactive and reactive control. Proactive control has costs and benefits. Following previous work (Griffiths et al., 2019; Lieder et al., 2018; Shenhav et al., 2017), we model the function of meta-control over proactive/reactive control as performing a cost-benefit analysis to determine whether the benefits of proactive control outweigh its costs. In this section, we develop a model of how people make meta-control decisions about i) whether to set an intention during the cue presentation and ii) whether to engage reactive control when the probe is present. To illustrate these meta-decisions, we return to the grocery shopping example from the introduction. In this example, the meta-control decision whether to set an intention to go shopping in the evening might be made when remembering the shortage of food at noon. This decision will be informed by the subjective importance of having more groceries, the expected increase in the probability of going grocery shopping if an intention is set, and the cost of setting the intention and remembering throughout the day. The meta-decision whether to boost or inhibit this intention occurs when the bus stop near the supermarket is announced. This decision will depend on whether the encountered situation matches the anticipated situation or not (e.g., supermarket closed or medical emergency), the cost of exerting control, and how likely it is that the intention will be enacted without boosting or inhibiting it. Finally, the meta-decision whether to engage reactive control would be made when a person who did not set an intention is reminded of their shortage of groceries when they see the supermarket. This meta-decision would be informed by the expected benefit of exerting control to stop daydreaming and hit the stop button and the effort that this would take. The meta-decision whether to engage reactive control might also occur when the presence inhibited their intention to go grocery shopping due to an unforeseen event (e.g., a medical emergency) and now faces a new situation that they were not prepared for.

We based the model of proactivity in the AX Continuous Performance Task on previous studies suggesting that reactive and proactive control are independent (Mäki-Marttunen et al., 2019a; Mäki-Marttunen et al., 2019b) and that people use different strategies in different trial types (Irlbacher et al., 2014). Furthermore, Mäki-Marttunen et al. (2019b) found that people’s performance in the AX-CPT decreases with the participant’s cognitive load, which they manipulated by varying whether there were 1, 2, or 3 letters that could instantiate the A-cue (load = 1, load = 2, and load = 3, respectively).

**Fig. 2:**
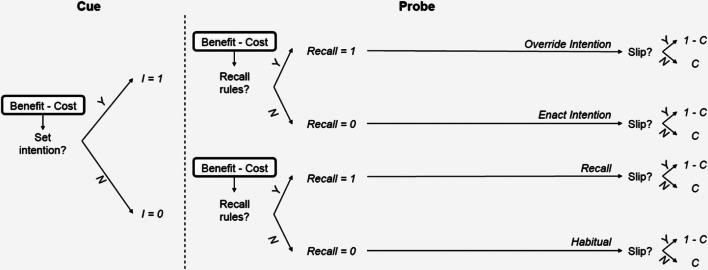
Our model of meta-control over proactive and reactive control. The variable *I* denotes whether the participant set an intention during the presentation of the cue. *C* is participant’s intended choice, that is the response that they will give unless their finger slips

According to our model the process of response selection includes two stages. The first stage begins with the presentation of the cue and the second stage begins with the presentation of the probe. Figure 2 illustrates the meta-control decisions that our model makes in the first stage and in the second stage, respectively. In the first stage, the meta-controller decides whether to proactively set an intention for how to respond to the probe (X or Y) while the cue (A or B) is being presented. In the second stage, the model’s behavior depends on whether an intention was set in the first stage. If no intention was set in the first stage, then the second stage decides whether to recall the rules and the cue and apply the rules or to react automatically to the probe based on habit. Regardless of whether an intention was set, the second stage decides whether to recall the rules or to enact the intention previously set. Thus, according to our model, there are several qualitatively different levels of proactivity in the AX-CPT and a person’s level of proactivity depends on two meta-control decisions that they make during each trial. In the remainder of this section, we detail our mathematical model of these meta-control decisions and the resulting response distributions.

Figure 2 illustrates how these meta-control decisions (i.e., intention setting and recalling the cue and rules) work together to determine the response to a cue-probe pair. According to our model, the probability of making the correct choice (*C* = 1) is the weighted average of the accuracies entailed by having set an intention (*I* = 1) versus not having set an intention (*I* = 0) given the model parameters *θ*, that is

1 $$ {\displaystyle \begin{array}{c}P\left(\mathrm{C}=1|\ \mathrm{probe},\mathrm{cue},\mathrm{load},\uptheta \right)=P\left(\mathrm{I}=1\ |\ \mathrm{cue},\mathrm{probe},\uptheta \right)\cdotp P\left(\mathrm{C}=1\ |\ \mathrm{I}=1,\mathrm{load},\mathrm{cue},\mathrm{probe},\uptheta \right)\\ {}+P\left(\mathrm{I}=0\ |\ \mathrm{cue},\mathrm{probe},\uptheta \right)\\ {}\cdotp P\left(\mathrm{C}=1\ |\ \mathrm{I}=0,\mathrm{load},\mathrm{cue},\mathrm{probe},\uptheta \right).\end{array}} $$

The model parameters *θ* = (u_+_, u_−_, u_Δ*t*_, λ, γ, δ) are summarized in Table 1 and will be explained one by one as we develop the model throughout the remainder of this section. Whether or not an intention is set (*P*(I = 1 | cue, probe, load, θ)) is determined by the meta-control decision made in response to the cue (Stage 1). Upon the presentation of the probe, the model makes a meta-control decision about whether or not to recall the rules (Stage 2). These meta-control decisions jointly determine the predicted accuracies in the scenario where an intention was set (*P*(C = 1 | I = 1, load, cue, probe, θ)) and the scenario where no intention was set (*P*(C = 1 | I = 0, load, cue, probe, θ)).

**Table 1:** Explanation of the model’s parameters *θ* = (*u*_+_, *u*_−_, *u*_*Δt*_, *λ*, *γ*)

Parameters	Explanation
*u*_+_	subjective utility of correctly detecting an AX trial.
*u*_−_	subjective utility of correctly reporting that a trial was not an AX trial.
*u*_Δ*t*_	subjective utility of responding quickly.
*λ*	intensity of the deleterious effect of cognitive load on controlled processing
*γ*	cost of setting an intention and maintaining it in working memory

### Stage 1: Deciding whether to proactively set an intention

To model the first stage (Fig. 2), we assume that, during the presentation of the cue, the person have the decision to set or not a behavioral intention, to respond affirmatively or not when the probe will be presented (e.g., set the intention to press “left” if a B cue is presented). We formalize this as a binary decision whether or not to proactively set an intention (*I* = 1 vs. *I* = 0) based on the cue (A or B) made according to an approximate cost-benefit analysis in which the meta-controller evaluates whether the expected value ($\mathbbm{E}$) of the benefits of engaging in proactive control outweighs its costs, that is whether

$$ \mathbbm{E}\left[\mathrm{Benefit}\left(I=1\right)\ |\ \mathrm{cue},{u}_{+},{u}_{-},{u}_{\Delta \mathrm{t}},\mathrm{load}\right]>\mathrm{cost}\left(I=1\right), $$

where the parameters *u*_+_ and *u*_−_ determine the utility or reward of making an accurate response specifically for AX or other trial types respectively and *u*_Δ*t*_ determines the utility of making a fast response, and *load* is the contextual load determined by the number of A-cues.

The benefit and the cost of proactively setting an intention (*I* = 1) are measured relative to the reward and cost of not setting an intention (*I* = 0). According to our model, the meta-control decision should be based on the expected benefit of proactive control given the information provided by the cue. Given that the cost-benefit analysis is approximate and that there is uncertainty about the relevant values, we model the probability that the meta-control system decides to engage in setting an intention as

2 $$ P\left(I=1|\mathrm{cue};\uptheta, \mathrm{load}\right)=\frac{\exp \left(\mathbbm{E}\left[\mathrm{Benefit}\left(I=1\right)\ |\ \mathrm{cue},{u}_{+},{u}_{-},{u}_{\Delta \mathrm{t}},\mathrm{load}\right]-\mathrm{cost}\left(I=1\right)\right)}{1+\exp \left(\mathbbm{E}\left[\mathrm{Benefit}\left(I=1\right)\ |\ \mathrm{cue},{u}_{+},{u}_{-},{u}_{\Delta \mathrm{t}},\mathrm{load}\right]-\mathrm{cost}\left(I=1\right)\right)}. $$

The cost-benefit analysis and the resulting probabilities of setting an intention in response to the A cue and the B cue are presented in the Supplementary Material.

**Fig. 3:**
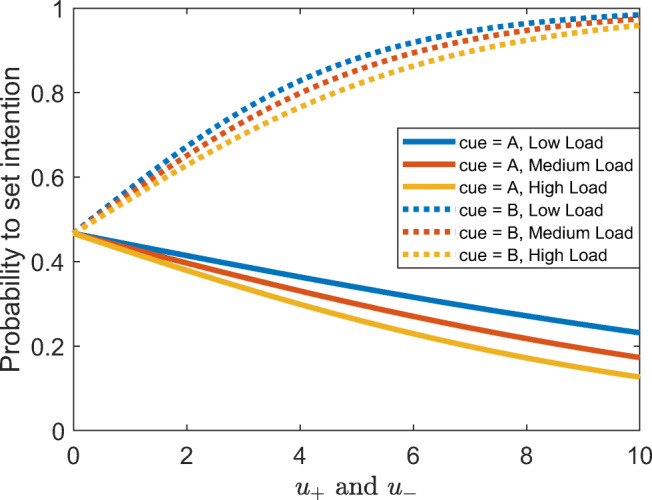
Probability of proactive intention setting in response to the cue (A vs. B) across low, medium, and high cognitive loads (load *=* 1, 2, and 3, respectively) depending on the reward for responding accurately (*u*_+_ and *u*_−_), assuming $\gamma =\frac{1}{3}$ , λ = 0.05, and *u*_Δ*t*_ = 0.2

Figure 3 shows our model’s predictions of the probability that people will proactively set an intention when they see a cue depending on the identity of the cue, cognitive load, and the reward for accuracy, *u*_+_ and *u*_−_, assuming that the cost of setting an intention and maintaining it in working memory is $\gamma =\frac{1}{3}$ and the utility of responding faster is *u*_Δ*t*_ = 0.2. Note that the addition of reward for accurate responses increases the probability to set an intention for B cues and decreases it for A cues because B cues are more informative of the correct response for that trial. Overall, it increases the model’s propensity towards proactive control. Thus, overall, proactivity should increase with reward and decrease with cognitive load. Our cost-benefit analysis model predicts that if people can switch between proactive and reactive control on a trial-by-trial level, then we should expect to see more proactive intention setting in response to B-cues than to A-cues. Furthermore, the cost-benefit analysis suggests that there could be interaction effects between cost and reward such that for very high and very low reward, the effect of load should be smaller than for intermediate levels of reward for B trials. Furthermore, the effect of cognitive load on intention setting should be higher on A-trials than on B-trials.

If the meta-control process has determined that an intention should be set, then intention setting proceeds by predicting the next stimulus and initiating the response to the predicted stimulus before it has even appeared. For instance, because the A is followed by an X in 87.5% of the time, proactive control will usually predict that the next stimulus will be an X and initiate an affirmative response to the upcoming probe. We therefore formally model proactive control as follows:

1. Make a prediction $\hat{s}$ about a future state (e.g., *S*_*t* + 1_) by sampling from the predictive model *ϑ* ($\hat{s}\sim P\left({S}_{t+1}|{S}_t={\hat{s}}_t;\vartheta \right)$) that has been learned through experience^1^ (Vul et al., 2014).
2. Plan one or more actions, *A*_*t* + 1_, to be taken in the predicted future state(s), $\hat{s}$, by maximizing the expected utility, $\mathbbm{E}$, of the resulting outcomes, *O*_*t* + 1_, (e.g., ${A}_{t+1}={\mathrm{a}\mathrm{rgmax}}_{\mathrm{a}}\mathbbm{E}\left[u\left({O}_{t+1}\right)|{S}_{t+1}=\hat{s},a\right]$). To simulated the AX-CPT, we model the utility of correctly detecting the AX stimulus by the free parameter *u*_+_, and we model the utility of correctly withholding that response in its absence by the free parameter *u*_−_, assuming that the utility of incorrect responses is 0.
3. Exert cognitive control to create the intention to execute the planned action(s) (e.g., take action *a* when the next stimulus appears), commit it to working memory, and actively maintain the memory of that intention.

For instance, upon seeing an A-cue in the AX-CPT with 70% AX trials and 10% AY trials, there is a 7 out of 8 chance that our model will predict that the probe will be an X. In that case, it will set the intention to give an affirmative response (e.g., “When the probe appears, I will click the button for reporting an AX-trial”). Alternatively, there is also a 1 in 8 chance that our model will predict that the A cue will be followed by a Y probe. In that case, it will set the intention to respond negatively (e.g., “When the probe appears, I will click the button for reporting that this is NOT an AX-trial”).

### Stage 2: Meta-control over the response to the probe

When participants encounter the probe, they may recall the cue and apply the rule for how to respond to the observed cue-probe (***c***_**recall**_ ***=*** **1**) or not (***c***_**recall**_ ***=*** **0**). We postulate that which of these two modes govern people’s responding is determined by a rational cost-benefit analysis (see Section 1.3 of the Supplementary Material). In brief, we model the meta-control decision whether or not to recall the cue and rules (reactive control) according to the soft-max decision rule

3 $$ P\left({c}_{\mathrm{recall}}=1|\mathrm{probe},{\mathrm{u}}_{+},{u}_{-},\upgamma, {\mathrm{u}}_{\Delta t},\mathrm{load}\right)=\frac{\exp \left(\mathbbm{E}\left[\mathrm{Benefit}\left({c}_{\mathrm{recall}}=1\right)\ |\ \mathrm{probe};{\mathrm{u}}_{+},{\mathrm{u}}_{-},{\mathrm{u}}_{\Delta t},\mathrm{load}\right]-\gamma \right)}{1+\exp \left(\mathbbm{E}\left[\mathrm{Benefit}\left({c}_{\mathrm{recall}}=1\right)\ |\ \mathrm{probe};{\mathrm{u}}_{+},{\mathrm{u}}_{-},{\mathrm{u}}_{\Delta t},\mathrm{load}\right]-\gamma \right)}, $$

where *γ* is the cost of recalling the cue and rules. The derivation of the benefit term is presented in the Supplementary Material. Since recalling and relying on the cue and the rules is a binary event, the probability of responding habitually is *P*(*c*_recall_ = 0| probe; u_+_, u_−_, γ, u_Δ*t*_, load) = 1 − *P*(*c*_recall_ = 1| probe; u_+_, u_−_, γ, u_Δ*t*_, load).

**Fig. 4:**
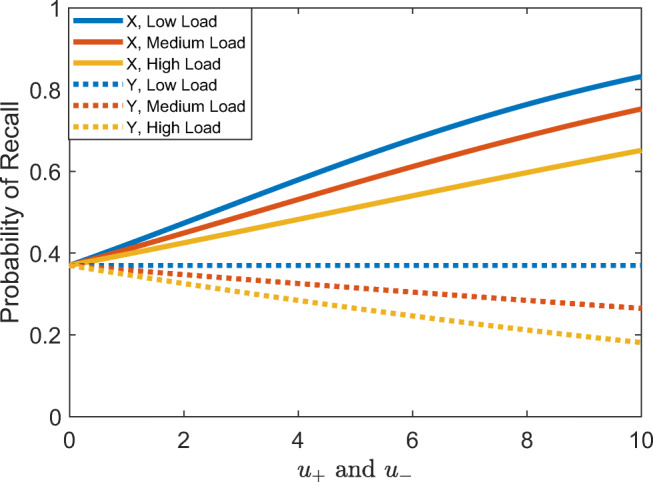
Probability of recalling the cue and rules in response to the probe (X vs. Y) across low, medium and high cognitive loads (load = 1, 2 and 3, respectively), depending on the reward for responding accurately (*u*_+_ and *u*_−_) assuming $\gamma =\frac{1}{3}$ , *λ* = 0.05, and *u*_*Δt*_ = 0.2

Figure 4 shows our model’s predictions of the probability of recalling the cue and rules across different rewards for being accurate (*u*_+_ and *u*_−_) for a fixed reward for being fast (*u*_Δ*t*_ = 0.2) and a fixed cost of holding information in working memory ($\gamma =\frac{1}{3}$). The probability of recalling in response to X probes is always predicted to be higher than for Y probes, since recalling the rules has no benefit when the automatic response would be correct as well. The model also predicts a higher probability of recalling for low contextual load conditions, since the probability of recalling the correct response decreases as the contextual load increases.

### Predicting people’s accuracy in the AX-CPT

To complete our model, we specify how likely the response is to be correct depending on whether or not an intention was set in response to the cue. The probability that the choice will be correct, *P*(*C* = 1), after an intention has been set (*I* = 1) is

4 $$ P\left(C=1\mid I=1,\mathrm{cue},\mathrm{probe},\mathrm{load},\theta \right)=P\left({c}_{\mathrm{recall}}=1\mid \mathrm{probe};{\mathrm{u}}_{+},{\mathrm{u}}_{-},\upgamma, {\mathrm{u}}_{\Delta t},\mathrm{load}\right)\cdot P\left({\mathrm{C}}_{\mathrm{R}}=1\mid \mathrm{cue},\mathrm{probe},\mathrm{load}\right)+P\left({c}_{\mathrm{recall}}=0\mid \mathrm{probe};{\mathrm{u}}_{+},{\mathrm{u}}_{-},\upgamma, {\mathrm{u}}_{\Delta t},\mathrm{load}\right)\cdot P\left({\mathrm{C}}_{\mathrm{I}}=1\mid \mathrm{cue},\mathrm{probe},\mathrm{load}\right)-\lambda \cdot \left(\mathrm{load}-1\right), $$

where *C*_*I*_ = 1 means that the intention was correct and the term −*λ* · (load − 1) models the deleterious effect of cognitive load on cognitive control with the parameter *λ* measuring the severity of this effect. The probability of the choice being correct given that an intention was set is given in Table 2. The probability that a correct choice will be made in the absence of an intention is

$$ P\left(\mathrm{C}=1|\ I=0,\mathrm{cue},\mathrm{probe},\mathrm{load},\uptheta \right)=\kern0.5em P\left({c}_{\mathrm{recall}}=1|\mathrm{probe};{\mathrm{u}}_{+},{\mathrm{u}}_{-},\upgamma, {\mathrm{u}}_{\Delta t},\mathrm{load}\right)\cdotp P\left({\mathrm{C}}_{\mathrm{R}}=1|\mathrm{cue},\mathrm{probe},\mathrm{load}\right)+P\left({c}_{\mathrm{recall}}=0|\mathrm{probe};{\mathrm{u}}_{+},{\mathrm{u}}_{-},\upgamma, {\mathrm{u}}_{\Delta t},\mathrm{load}\right)\cdotp P\left({\mathrm{C}}_{\mathrm{M}}=1|\mathrm{cue},\mathrm{probe},\mathrm{load}\right)-\lambda \cdotp \left(\mathrm{load}-1\right), $$

where C_R_ = 1 means successfully recalling and applying the rule and C_M_ = 1 is the accuracy of probability matching. The probabilities of the choice being correct given successfully recalling and applying the rule are given in Table 2.

**Table 2:** Probability that the response is correct if it is driven by an intention (2^nd^ column), by automaticity based on probability matching (3^rd^ column), or reactive control (4^th^ column) depending on the trial type. In this example there are 70% AX trials, 10% AY trials, 10% BX trials, and 10% BY trials. The first term of the accuracy of proactive control (e.g., $\frac{7}{8}$) is the probability that the person predicted the more likely probe when they set their intention. The second term is the loss in accuracy due to cognitive load. *load* is the intensity of the cognitive load given by the number of different letters that could serve as the A-cue (i.e., 1, 2*,* or 3).

Cue, probe	Accuracy of proactive control *P*(C_I_ = 1| cue, probe, load)	Accuracy of automaticity *P*(C_M_ = 1| cue, probe)	Accuracy of reactive control *P*(C_R_ = 1| cue, probe, load)
AX	$\frac{7}{8}-\lambda \cdotp \left(\mathrm{load}-1\right)$	$P\left( AX|X\right)=\frac{7}{8}$	1 − *λ* · (load − 1)
AY	$\frac{1}{8}-\lambda \cdotp \left(\mathrm{load}-1\right)$	*P*(*AX*′| *Y*) = 1	1 − *λ* · (load − 1)
BX	1 − *λ* · (load − 1)	$1-P\left( AX|X\right)=\frac{1}{8}$	1 − *λ* · (load − 1)
BY	1 − *λ* · (load − 1)	*P*(*AX*′| *Y*) = 1	1 − *λ* · (load − 1)

Furthermore, we assume that a random error, in which the motor slips cause the button press to be incorrect, *R* = 0, even if the participant’s choice was correct (*C* = 1) and vice versa. We therefore model the probability that the button press is correct as

5 $$ {P}_{\mathrm{model}}\left(\mathrm{R}=1|\mathrm{cue},\mathrm{probe},\uptheta \right)=P\left(\mathrm{C}=1|\mathrm{probe},\mathrm{cue},\uptheta \right)\cdotp \left(1-{p}_{\mathrm{slip}}\right)+P\left(\mathrm{C}=0|\mathrm{probe},\mathrm{cue},\uptheta \right)\cdotp {p}_{\mathrm{slip}}. $$

Finally, we assume that the probability that the participant’s hand slips is equal to the error rate in BY trials, that is *p*_slip_ = 0.0125 for the Mäki-Marttunen et al. (2019a) dataset. This model formalizes the key assumption of the dual mechanisms of control (DMC) framework that people make two independent meta-control decisions about whether to engage proactive control and whether to engage in reactive control according to a rational cost benefit analysis. We therefore refer to this model as our DMC model (*m*_DMC_).

### Alternative meta-control mechanisms

#### Inhibition of prepotent intentions

Recent physiological data and reaction time data suggest that there might be an additional control mechanism influencing people’s responses in the AX-CPT, namely the inhibition of prepotent responses (Mäki-Marttunen et al., 2018; Mäki-Marttunen et al., 2019b). This means that a participant might see an A cue and set the prepotent intention to report an AX trial in anticipation of an X probe and then inhibit that intention when they see the Y probe. In support of this view, the pupillometry findings of Mäki-Marttunen et al. (2018) and the Locus Coeruleus and dlPFC activation found by Mäki-Marttunen et al. (2019a, 2019b) support the involvement of inhibitory control in the AX-CPT. Furthermore, the especially long response times on AY trials might suggest that participants sometimes override their intention to report an AX pair when they see the Y probe (Mäki-Marttunen et al., 2018; Mäki-Marttunen et al., 2019b). For these reasons, we developed an extension of the model illustrated in Fig. 2 that includes an additional meta-decision about whether to inhibit the proactively set intention when it encounters the probe.

As illustrated in Fig. 6a, this model assumes that if people have proactively set an intention in response to the cue (A or B) then the control system may boost or inhibit reactive control in response to the probe (X or Y). For instance, if the participant set the intention “Click the button for AX trials when the probe appears” in response to the A-cue, then they might inhibit this incorrect intention when they see the Y probe. We assume that the probability that a participant will do this increases with the benefits of being correct and decreases with the cost of control. Conversely, when the participant encounters the anticipated X probe, they might boost their intention to report an AX trial to further increase their probability of being correct if they are highly motivated to be fast and accurate. Furthermore, inhibition might be especially important on no-go trials where a third type of probe signals that the response should be withheld.

We model this decision as the specification of a control signal *c* ∈ [−*c*_0_, 1 − *c*_0_] that shifts the probability that the decision will be determined by the proactively formed intention away from its default probability of *c*_0_ = 0.7. A positive control signal boosts the effect of the proactively formed intention, whereas a negative control signal inhibits it. We further assume that cognitive load has an interference cost, *λ*, that affects the probability to successfully inhibit a proactively set intention. Formally, we assume that the probability that the intention will be inhibited is *P*(Inhibit = 1| *c*) = (1 − (c_0_ + *c*)) · (1 − *λ* · load).

According to our model, the intensity of the control signal *c* is chosen according to a cost-benefit analysis. This cost-benefit analysis assumes that the control signal intensity *c* linearly interpolates between the expected performance of responding with versus without the intention (see Section 1.2 of the Supplementary Material). Following previous work, we model the cost of inhibition as an exponential function of the absolute value of the control signal intensity, in which *δ* is the control cost parameter that determines how quickly the cost of control increases with the absolute value of the control signal, that is

$$ \mathrm{cost}\left(c;\delta \right)=\exp \left(\delta \cdotp |c|\right)-1. $$

Therefore, the optimal control signal *c*^⋆^ is

$$ {c}^{\star}\left(\mathrm{probe},{u}_{+},{u}_{-},{\mathrm{u}}_{\Delta t},\delta \right)=\arg \underset{\mathrm{c}}{\max}\left(\mathbbm{E}\left[\mathrm{Benefit}(c)|\ \mathrm{probe},{\mathrm{u}}_{+},{u}_{-},{\mathrm{u}}_{\Delta t},\mathrm{load}\right]-\mathrm{cost}\left(c;\delta \right)\right). $$

Assuming that the cognitive control system chooses the optimal control signal, the probability that the person will inhibit the intention is

6 $$ P\left(\mathrm{Inhibit}|\mathrm{probe},\mathrm{load},\theta \right)=\left(1-\left(0.7+{c}^{\star}\left(\mathrm{probe},{\mathrm{u}}_{+},{u}_{-},{\mathrm{u}}_{\Delta t},\delta \right)\right)\right)\cdotp \left(1-\lambda \cdotp \mathrm{load}\right). $$

**Fig. 5:**
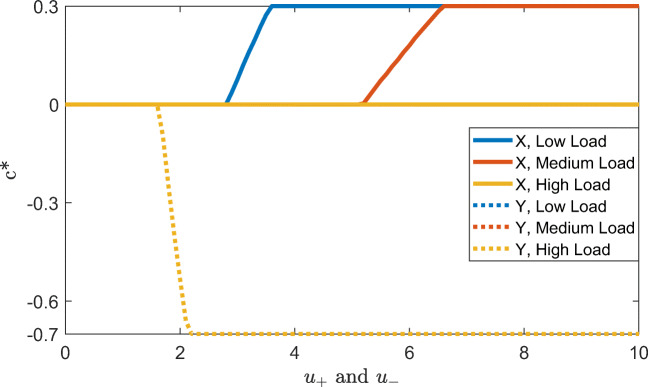
Optimal control signal intensity depending on the reward for responding accurately (u_+_ and u_−_) across low, medium and high cognitive loads (load = 1, 2, and 3, respectively) assuming $\updelta =\frac{1}{2}$,$\upgamma =\frac{1}{3}$, λ = 0.05, and u_Δt_ = 0.2

Figure 5 shows the optimal control signal intensity as a function of the subjective utility of correct and fast responses depending on the currently presented probe. The plot shows that, given a sufficiently high reward, the model boosts the effect of the proactively set intention for X probes and inhibits the intention for Y probes. The optimal control signal intensity remains zero for a wide range of values for *u*_+_ = *u*_−_ = *u*_*Δt*_ for which the cost of control outweighs any potential benefits.

One can think of this second step as modulating the intensity of the effect of setting a proactive intention in the first step. The control signal can diminish the increase in proactivity or enhance it further. Regardless of the chosen control signal, the level of proactivity should always be higher when a proactive intention was formed in the first step than when it was not.

The inclusion of this additional meta-control decision leads to the following equation for the probability of making a correct choice:

$$ P\left(C=1\ \right|\ I=1,\mathrm{cue},\mathrm{probe},\mathrm{load},\theta \left)=\left(1-P\left(\mathrm{Inhibit}\ |\ \mathrm{probe},\theta, \mathrm{load}\right)\right)\cdotp P\left({\mathrm{C}}_{\mathrm{I}}=1\right|\ \mathrm{cue},\mathrm{probe},\mathrm{load}\right)+P\left(\mathrm{Inhibit}\ |\mathrm{probe},\theta, \mathrm{load}\right)\cdotp P\left(\mathrm{C}=1|I=0,\mathrm{cue},\mathrm{probe},\mathrm{load},\theta \right)-\lambda \cdotp \left(\mathrm{load}-1\right). $$

#### Models according to which proactive and reactive control are mutually exclusive

**Fig. 6:**
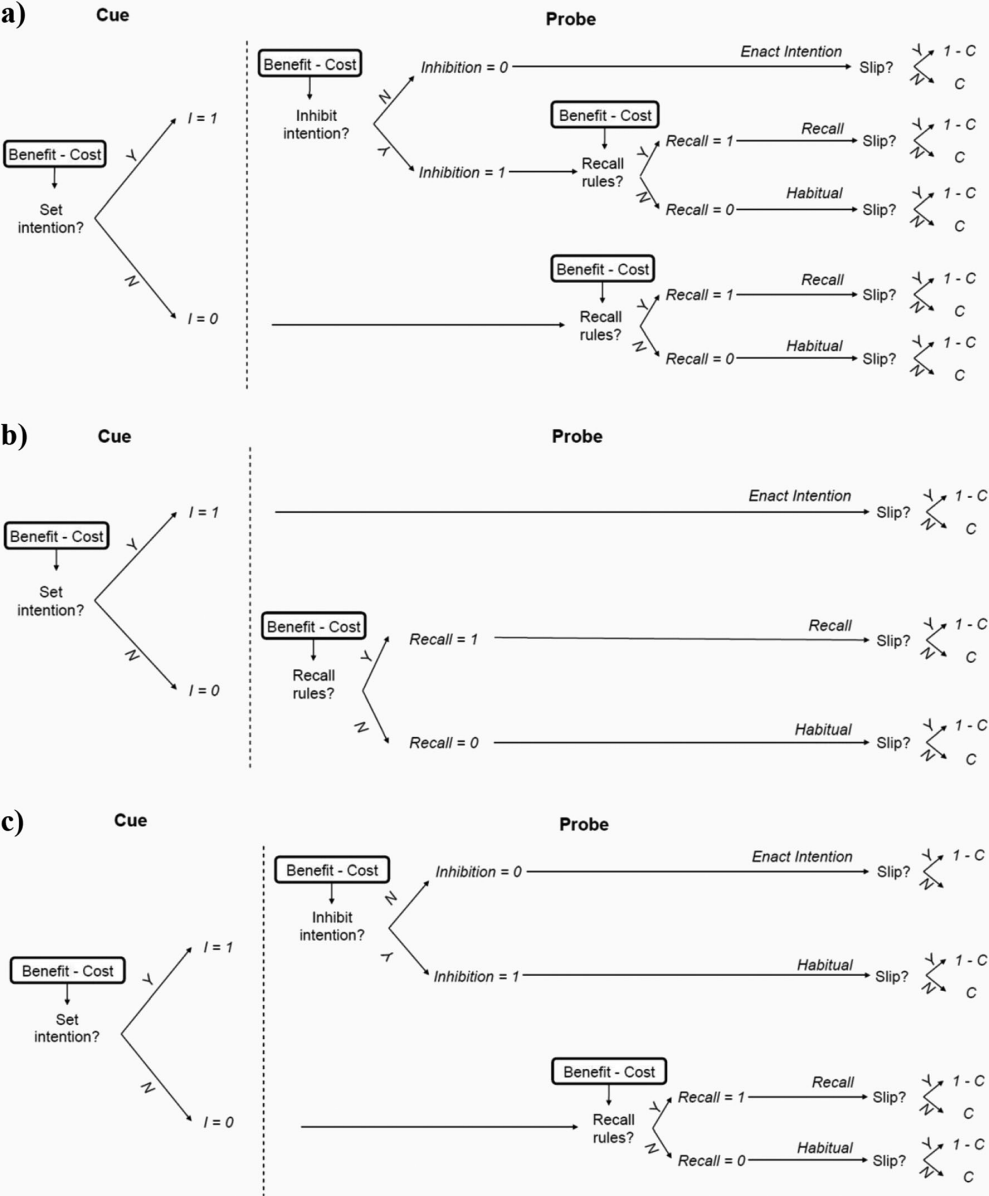
Alternative models. **a** Extended meta-control model according to which intentions can be inhibited and reactive control is not considered when the person has an active intention. **b** Simpler model according to which the meta-control decision about recalling the rules (reactive control) is only made when no intention was set (exclusivity model without inhibition). **c** An extension of the model shown in Panel b according to which the proactively set intention can be inhibited (exclusivity model with inhibition)

A key assumption of the model illustrated in Fig. 2 is that people can always override the intention that they have set in response to the cue (proactive control) by invoking reactive control in response to the probe (reactive control). According to the extended meta-control model illustrated in Fig. 6a, this is possible only when the proactively set intention is inhibited first. A third alternative is that proactive control and reactive control might be mutually exclusive in the sense that people have choose between one or the other but cannot use both. The mutual exclusivity models illustrated in Fig. 6b-c formalize this assumption in two different ways. According to the mutual exclusivity model without inhibition (Fig. 6b) the participant either sets an intention and then enacts it (proactive control) or does not set an intention and then chooses whether to recall the rules (reactive control). According to the mutual exclusivity model with inhibition (Fig. 6c), a participant who has set an intention can choose to inhibit it, but when they do their response is determined by their habits (no control).

### Summary

Our model postulates that meta-control over proactivity is governed by two rational cost-benefit tradeoffs that governed whether intentions are set proactively, and whether the cue and rules are recalled and followed. The first meta-decision is made based on the cue, whereas the second one is made based on the probe. All meta-decisions are informed by the incentives for speed and accuracy, cognitive load, and the cost of control. Before testing the predictions of the meta-control model, we will present and test the assumptions about the proactive process.

## Results: Explaining variability of control in the AX Continuous Performance Task

Our meta-control model of the variability in proactive behavior comprises three components: 1) its assumptions about which meta-control decisions govern the variation in people’s proactivity, 2) its predictions of the accuracies given the outcomes of those meta-control decisions, and 3) its predictions of the meta-control decisions about intention setting and recalling the rules. In this section, we leverage previously published empirical data to examine each of these three components in turn. Each time we use the method described in Section 2 of the Supplementary Material to fit the parameters of our models to the data.

### Testing alternative theories of meta-control over proactivity

To identify which of the four models introduced above best describes meta-control over proactivity in the AX-CPT, we performed a formal model comparison. The distinguishing feature of two of our models is that they inhibit a previously set intention. This feature is most relevant when the task includes no-go trials. We therefore compared the four models on the data set from Experiment 2 by Gonthier et al. (2016). Assuming that the mechanisms of meta-control might differ across participants, we performed one formal quantitative model comparison for each individual participant. To select between the models, we used the Bayesian Information Criterion (Schwarz, 1978). As summarized in Table 3, the results of our model comparison strongly support the DMC model according to which people make two independent meta-control decisions about invoking proactive and reactive control respectively. In fact, the DMC model was the best model for all 92 participants when compared to the extended model and the exclusivity model with inhibition and for 73.9% of the participants compared with the exclusivity model without inhibition.

**Table 3:** Results of the participant-level (N = 92) model comparison on the data set by Gonthier et al. (2016) between the DMC model (m_DMC_) shown in Fig. 2 and the alternative models (m_alternative_) shown in Fig. 6a-c. The model comparisons were performed based on $\Delta \mathrm{BIC}=\mathrm{BI}{\mathrm{C}}_{{\mathrm{m}}_{\mathrm{DMC}}}-\mathrm{BI}{\mathrm{C}}_{{\mathrm{m}}_{\mathrm{alternative}}}$.

	Extended model (Fig. 6a)	Exclusivity without inhibition (Fig. 6b)	Exclusivity with inhibition (Fig. 6c)
% participants whose data is best explained by this model:	0%	26.1%	0%
% participants for whom the evidence for *m*_DMC_ over *m*_alternative_ is			
very strong (ΔBIC > 10)	7.6%	**-**	8.7%
strong (6 < ΔBIC < 10)	50.0%	**-**	46.7%
positive (2 < ΔBIC < 6)	40.2%	10.9%	41.3%
weakly positive (0 < ΔBIC < 2)	2.2%	63.0%	3.3%
weakly negative (−2 < ΔBIC < 0)	**-**	26.1%	**-**
negative ( −6 < ΔBIC < − 2)	**-**	**-**	**-**
strongly negative (−10 < ΔBIC < − 6)	**-**	**-**	**-**

### Testing the model’s predictions about the effects of meta-control

**Fig. 7:**
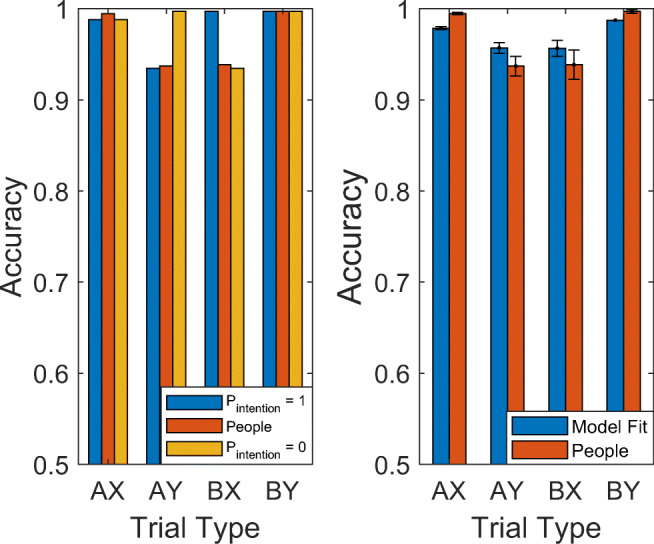
The plot on the left compares the accuracies of people to the accuracies that our model predicts if people were to always set intentions (*p*_intention_ = 1) or never set intentions (*p*_intention_ = 0) for low contextual load (load = 1), and assuming and *p*_*recall*_= 0.93. On the right, mean accuracy when fitting the model across trials of the no reward and low cognitive load condition for each participant with *p*_*intention*_, (*M* = 0.58, *SD* = 0.30) and *p*_*recall*_ (*M* = 0.93, *SD* = 0.09) as free parameters. All error bars in this article convey the standard error of the mean

If our model of how people set intentions and how intentions affect behavior is correct, then people’s response frequencies on AY trials and BX trials should be a weighted average of the response frequencies that our model predicts for the case when an intention is set (proactive control) and the case when no intention is set (no proactive control). As illustrated in Fig. 7a, the data from Mäki-Marttunen et al. (2019a) confirmed this prediction. This suggests that we can understand the average response frequencies across people and experimental conditions as a mixture between these two modes of control. To gauge the relative contributions of proactive control and reactive control, we used maximum likelihood estimation to fit the parameters *p*_intention_, and *p*_recall_ to the accuracies of individual participants. As Fig. 7b shows, this model achieves an impressively good fit with the most likely *p*_intention_ = 0.58. This suggests that, on average, people engage in proactive control in about 58% of the trials of this task. Consistent with the dual mechanisms of control framework (Braver, 2012), we found substantial interindividual differences in the propensity to engage proactive control. Concretely, individual participants’ propensities to set intentions ranged from 0.0001% to 100% (*M* = 0.58, *SD* = 0.30) Participants’ propensity to engage in reactive control appeared to be less variable. That is, people’s propensity to recall the rules when the encountered the probe without a prepared intention ranged from 54% to 100% with a mean of 93% and a standard deviation of *9%* (*M* = 0.9281, *SD* = 0.09)*.*

### Testing the model’s predictions about when people set intentions

To test whether our model can capture when people set intentions, we simulated the experiments conducted by Mäki-Marttunen et al. (2019a), Redick (2014), and Gonthier et al. (2016). Each time, we compare how often people should set an intention or recall the rules according to our model to how often they actually do. To estimate how often people engage in these processes, we estimate the parameters *p*_intention_ and *p*_recall_ of a simple measurement model from people’s responses. This measurement model describes participants’ response probabilities, *R*, by

$$ {P}_{\mathrm{measurement}}\left(R|\mathrm{cue},\mathrm{probe},\mathrm{load},\theta; m\right)=\sum \limits_{\mathrm{I},\mathrm{recall}\in \left\{0,\kern0.5em 1\right\}}P(I)\cdotp P\left(\mathrm{recall}\right)\cdotp P\left(R|\mathrm{cue},\mathrm{probe},\theta, \mathrm{I},\mathrm{recall},\mathrm{load};m\right),\kern0.5em $$

where *P*(*I* = 1) = *p*_intention_, *P*(Recall = 1) = *p*_recall_, and *m* is our model of how proactive and reactive control affect people’s accuracies.

#### Prediction 1: Effect of incentives on proactivity

**Fig. 8:**
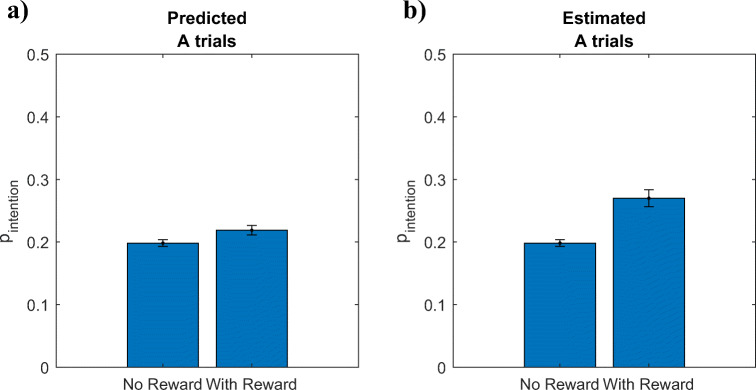
Predicted and estimated effects of incentivizing fast, accurate responses on the probability of proactive intention setting

The reward condition of the experiment reported by Mäki-Marttunen et al. (2019a) incentivized fast accurate responses in a standard AX-CPT task with 70% AX trials, 10% AY trials, 10% BX trials, and 10% BY trials. Assuming that these incentives increase the subjective reward for identifying AX pairs (u_+_) and responding faster (*u*_Δ*t*_) by one unit, Equation 2 of our model predicts that incentivized participants should be more likely to proactively set intentions than unincentivized participants on A-trials (Figs. 8a and 12) as well as on B-trials and become more proactive overall. Concretely, our model predict that incentives should increase the probability of intention setting on A trials from 20% to 22% and increase the probability of intention setting on B trials from 80% to 84%. To test our meta-control model’s prediction about the probability of proactive intention-setting, we used the maximum likelihood estimation method described in Section 2 of the Supplementary Material to estimate the relative frequencies with which participants proactively set intentions (*p*_intention_) separately to A-trials and B-trials, respectively. As shown in Fig. 8b, the maximum likelihood estimates showed that the incentives increased the probability of intention setting on A-trials from 20% (*SD* = 0.06) to 27% (*SD* = 0.15; *t*(264) = 4.91, *p* < 0.001). The incentives’ effect on the probability of intention setting on B-trials was very close to the model’s prediction (80% (SD = 0.23) vs. 83% (SD = 0.22)), but this effect was not statistically significant (*t*(264) = 0.97, *p* = 0.33).

#### Prediction 2: Effect of incentives on accuracy on AY trials

**Fig. 9:**
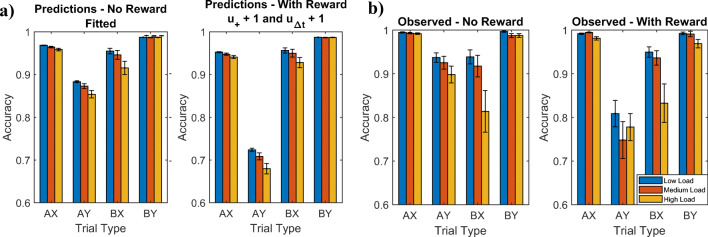
Comparison of predicted versus observed effects of incentivizing fast, accurate responses in the behavioral experiment by Mäki-Marttunen et al. (2019a). **a** Prediction of our meta-control model. **b** Human performance

To predict the effect of incentivizing participants to rapidly generate correct responses, we first fitted the parameters of our meta-control model to individual participants’ accuracies in the nonreward condition of the experiment by Mäki-Marttunen et al. (2019a) using the method described in Section 2 of the Supplementary Material and then used our model to simulate what their accuracies would have been if each participant’s subjective utilities for responding fast (*u*_Δ*t*_) and correctly identifying AX pairs (*u*_+_) had been one unit higher. As illustrated in Fig. 9a, our model predicts that an increased reward for accurately identifying AX pairs (u_+_) and responding faster (*u*_Δ*t*_) should have a negative effect on people’s performance on AY trials (Fig. 9a). Consistently with this prediction, Mäki-Marttunen et al. (2019a) found that reward incentives decreased people’s performance on AY trials (78% vs. 92%, *t*(264) = −6.43, one-tailed *p* < 0.0001; Fig. 9b). As our model had predicted (see Fig. 9a), this inverted the relationship between people’s performance on AY trials versus AX trials. That is, people’s average performance in the experiment by Mäki-Marttunen et al. (2019a) was higher on AY trials than on BX trials in the no-reward condition (92% vs. 89%; *t*(260) = 1.41, *p* = 0.0799), but the opposite was the case in the reward condition (78% vs. 91%; *t*(268) = −4.90, *p* < 0.0001; Fig. 9b).

#### Prediction 3: Effect of the relative frequency of AX trials on proactive intention setting

**Fig. 10:**
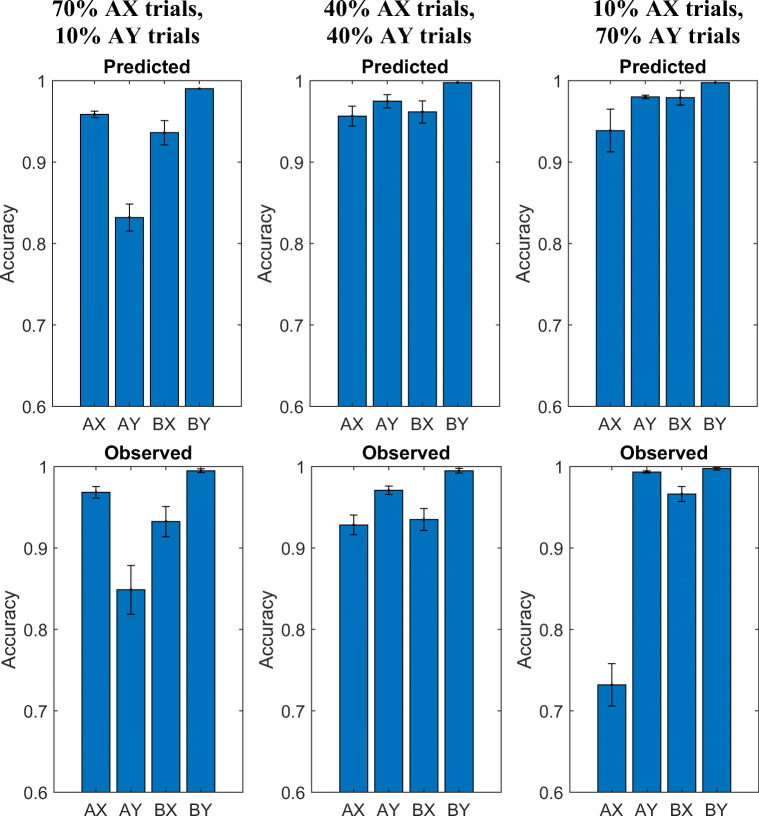
Mean predicted (upper) and observed (lower) accuracies for Redick (2014) datasets

As illustrated in Fig. 14a, our meta-control model predicts that people’s propensity to engage proactive control should decrease as the relative frequency with which an A is followed by an X drops to 50%. This is intuitive because the proactively set intention in the AX-CPT is correct only when the A is followed by an X. Because proactive control increases the frequency of errors on AY trials, decreasing the frequency of AX trials therefore should increase participants’ accuracy on AY trials, as illustrated in the top row of Fig. 10. As shown in the bottom row of Fig. 10, the experiments reported in Redick (2014) confirmed this prediction. Furthermore, people remain highly accurate on AY trials as their frequency exceeds the frequency of AX trials. According to our model, this is because people will then prepare to respond to Y when they see the A.

#### Prediction 4: Effect of no-go trials

**Fig. 11:**
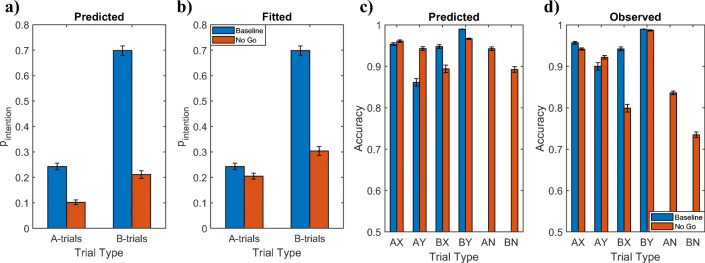
**a** Predicted p_intention_ for baseline and no-go conditions. **b** Fitted accuracies for baseline and no-go conditions. ***c*** Predicted accuracies for the conditions with versus without no-go trials suing the maximum likelihood estimates for the baseline condition and only changing the relative frequency of the trial types for no-go condition. ***d*** Observed accuracies in Experiment 2 by Gonthier et al. (2016).

To predict the effect of adding no-go trials on people’s performance on AX, AY, BX, and BY trials, we fitted our model’s parameters to data from a standard AX-CPT without no-go trials (i.e., the control condition of Experiment 1 from Gonthier et al., 2016) and then used the estimated parameters to simulate how the accuracies should change when no-go trials are added (i.e., Experiment 2 of Gonthier et al., 2016). If a participant set an intention on a no-go trial, then they either have to inhibit that intention in response to the no-go probe, which costs time and effort, or accept the penalty for giving an incorrect response. This is why the possibility of no-go trials reduces the expected benefit of intention setting and reduces its expect cost. Therefore, our rational model predicts that adding no-go trials should decrease proactive intention setting in response to both A cues and B cues (Fig. 11a). Figure 11b shows that this is indeed the case. Because of this effect our model also predicts that no-go trials should increase people’s accuracy on AY trials and decrease their accuracy on BX and BY trials (Fig. 11c). As Fig. 11d shows, Experiment 2 from Gonthier et al. (2016) confirmed this prediction.

#### Prediction 5: Intention setting on BX versus AY trials

**Fig. 12:**
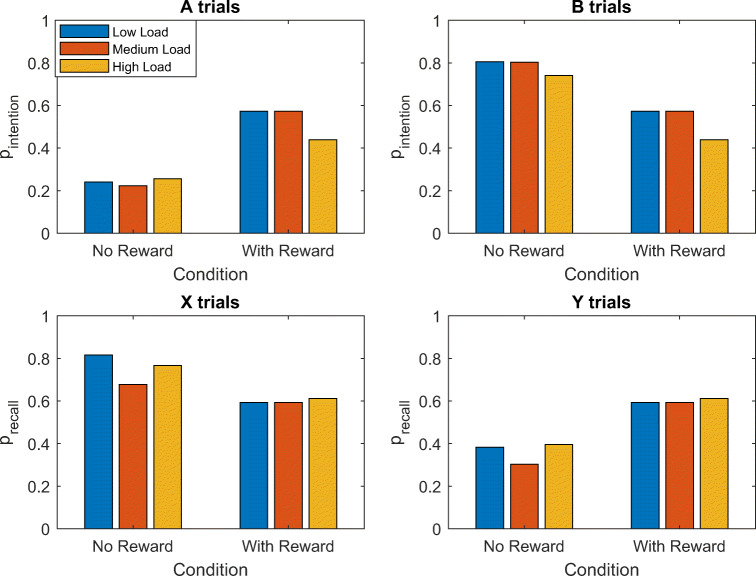
Estimates of the probability to set an intention and the probability of recalling the rules by trial type and experimental condition. These estimates were obtained by fitting our meta-control process model to all accuracies of individual participants using the method described in Section 2 of the Supplementary Material.

According to our meta-control model, on BX trials the probability to set an intention should be higher and responses should be the faster than on AY trials according to our model. This is because B cues induce the highest probability of engaging proactive control and X probes boost proactive control, whereas A cues induce a lower probability of engaging proactive control and Y probes trigger an inhibition of the proactively prepared intention (Fig. 12).

Confirming our model’s prediction, the reaction times reported by Mäki-Marttunen et al. (2019a) are significantly lower for BX trials than for AY trials across all six experimental conditions (*t*(530) = −3.67, *p* < 0.001), and the average probability of intention setting was significantly larger for BX trials (*M* = 0.7846; *SD* = 0.2138) than for AY trials (*M* = 0.2630, *SD* = 0.1418; *t*(530) = 33.1610, *p* < 0.0001).

#### Prediction 6: Effect of contextual load on accuracy

**Fig. 13:**
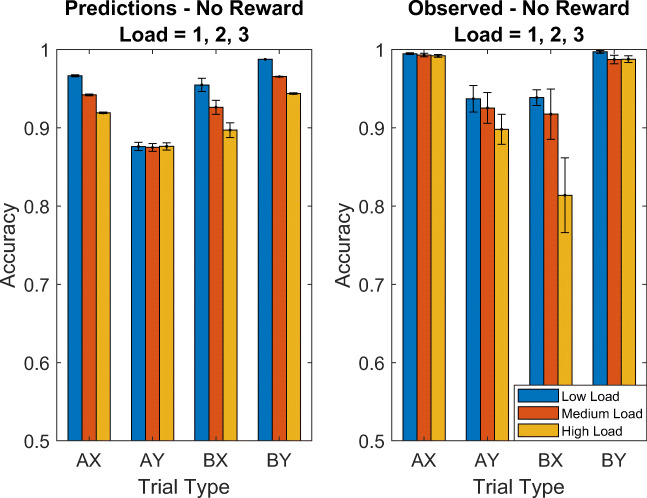
Predicted effects of contextual load on accuracy

To predict the effect of contextual load on accuracy, we fitted our model’s parameters the individual participants’ response in the condition of the experiment by Mäki-Marttunen et al. (2019a) that had a contextual load of 2 while constraining *λ* to be at least 0.025. We then simulated how the accuracies should change when the contextual load is increased to 3 or decreased to 1. We performed this procedure separately for the reward condition and the no reward condition. As shown in Fig. 13, our model predicted that cognitive load should reduce people accuracy on AX trials, BX trials, and BY trials, but not on AY trials.

Confirming this prediction, Mäki-Marttunen et al. (2019b) found that on AX, BX, and BY trials people’s accuracy was significantly lower for high cognitive load than for low cognitive load and Mäki-Marttunen et al. (2019a) found this effect for AX and BX trials when comparing the high load condition to either the low load condition or the intermediate load condition. We found that to obtain this prediction with our model, it is critical to assume that there is a direct interference of load on the accuracy of controlled processing (*λ* > 0). As our model predicted, no effect of load was found for AY trials. The model-based estimates shown in Fig. 12 suggest that the effect of cognitive load on accuracy might be mediated by a reduction in intention setting in the high load condition.

## Discussion

In this article, we introduced and validated a computational level theory of the meta-control decisions that determine people’s level of proactivity in the AX-CPT. In doing so, we have instantiated the dual mechanisms of control framework (Braver, 2012) in terms of a precise computational model of the meta-control decisions that drive variability in cognitive control. Our model predicts when people engage proactive control, reactive control, or both. Our model captures that proactivity involves setting intentions based on predictions about the future, allocating control to those intentions when opportunities arise, and inhibiting competing automatic responses. The basic idea of our model is that proactivity is governed by the allocation of control according to a rational cost-benefit analysis. Empirical data from previous experiments supported numerous predictions of our model, including its predictions about the effects of incentives, contextual load, adding no-go trials, and changing the ratio of AX trials to AY trials. This suggests that our model is a promising step towards unraveling the computational mechanisms of proactivity. Understanding proactivity, in turn, is an important step towards understanding what it takes to live a successful life and how we can assist people in this challenging process (Lieder & Prentice, 2020).

### Implications for our theoretical understanding of proactivity in the AX-CPT

Our model formalizes central ideas of the Dual Mechanisms of Control framework (Braver, 2012) in terms of rational tradeoffs between the costs of exerting cognitive control and its benefits (Lieder & Griffiths, 2020; Shenhav et al., 2017). Its success in predicting the effects of adding incentives and increasing cognitive load (Mäki-Marttunen et al., 2019b), reducing the frequency of AX-trials (Redick, 2014), adding no-go trials (Gonthier et al., 2016), therefore lends some support to viability of those theoretical assumptions. Most strikingly, our model’s generalization at predicting the effects of changing the statistical structure of the task by reducing the frequency of AX trials (Redick, 2014) or adding no-go trials (Gonthier et al., 2016) supports its central assumption that people engage proactive control according to a rational cost-benefit analysis. This lends further support to the Dual Mechanisms of Control framework (Braver, 2012), the expected value of control theory (Shenhav et al., 2013), and the theory of resource-rationality (Lieder & Griffiths, 2020).

Our model comparisons strongly supported a meta control model in which proactive and reactive control are independent and can both occur on the same trial. This version of our model makes exactly two independent meta-control decisions: one about whether or not to engage proactive control and a second one about whether or not to engage reactive control. Supporting the view that proactive control and reactive control are independent, the meta-control decision about reactive control is always made regardless of whether an intention was previously set. It decides whether to recall the rules based on a cost-benefit analysis that only considers the probe. If the rules are recalled then whatever mechanisms would have determined the person’s choice otherwise, be it a proactively set intention or automaticity, will be inhibited and overridden by reactive control. Supporting the view that the inhibition of prepotent intentions is an important part of reactive control, the data favored the recall-override model over an alternative simplified model that does not allow for the inhibition of proactively set intentions. In addition, the simulation results summarized in Fig. 5 of the Supplementary Material show that proactive control and reactive control are two complementary mechanisms of proactivity that are both needed to capture people’s capacity for proactive goal-directed behavior.

### Future Directions

**Fig. 14:**
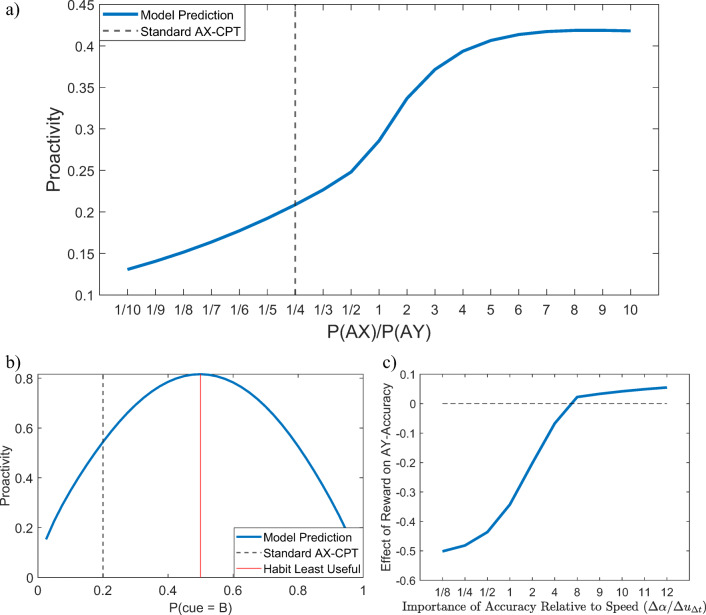
Model predictions to be tested in future work*.* The vertical dashed lines in **a** and **b** mark the values corresponding to a standard AX-CPT with 70% AX trial, 10% AY trials, 10% BX trials, and 10% BY trials. **a** Our model predicts an increase of proactivity with the frequency of AX trials relative of the frequency of AY trials. In this simulation there are 10% BX trials, 10% BY trials, and AX and AY trials jointly make up the remaining 80% of the trials. A ratio of 1 means that there are 40% AX trials and 40% AY trials. A ratio of 20 means that there are 3.5% AY trials and 76.5% AX trials. The model parameters are those used to simulate the reward condition. Proactivity is measured according to Equation 13 in the Supplementary Material. **b** Our model predicts an inverse-U-shape relationship between the proportion of B-cues (*p*_*B*_) when the relative frequencies of AX, AY, BX, and BY trials are $\frac{7}{8}\cdotp \left(1-{p}_B\right),\frac{1}{8}\cdotp \left(1-{p}_B\right),\frac{7}{8}\cdotp {p}_B$, and $\frac{1}{8}\cdotp {p}_B$ respectively. The model predictions suggest that people should be most proactive when habits are least useful. ***c***) For a sufficiently high reward (4 times the subjective value of getting it correct without any reward) our model predicts that the effect of reward on people’s performance on AY trials should switch from decreasing people’s performance to increasing people’s performance as the emphasis of the reward criterion shifts from speed to accuracy

Our model allows us to derive a number of predictions that go beyond the phenomena studied by Mäki-Marttunen et al. (2019a), Mäki-Marttunen et al. (2019b), Gonthier et al. (2016), and Redick (2014). These predictions can guide the design of future experiments. In general, our rational model of meta-control predicts that proactivity increases with situational factors and personal characteristics that make proactivity more beneficial and decreases with situational factors and personal characteristics that make it costlier and less beneficial. The value of proactivity increases with the predictability of the environment. Therefore, our model predicts that for AX frequencies in-between the extremes used by Redick (2014) people should gradually become less proactive as more AX trials are converted into AY trials. Conversely, and this has not been explored yet, people should become more proactive when the frequency of AX trials is increased beyond 70% or the frequency of AY trials is decreased below 10% (Fig. 14a). Based on this prediction, proactive intention setting should be most frequent in response to a cue that is always followed by the same probe and inverts the required response to that probe. Proactivity also becomes more valuable as reflexive responding to the probe becomes less effective. Reflexive responding to the X probe is least effective when both responses are correct equally often. This is the case when the frequency of BX trials equals the frequency of AX trials. Our model therefore predicts an inverse-U shaped effect of the proportion of B-cues on proactivity when the ratio of AX trials to AY trials and the ratio of BX trials to BY trials is held constant (Fig. 14b). Furthermore, when the incentives or instructions emphasize accuracy over speed, then the reward manipulation would have the opposite effect on people’s performance on AY trials than the one observed by Mäki-Marttunen et al. (2019a); that is, as the emphasis of the reward criterion shifts from speed towards accuracy, the effect of reward on people’s performance on AY trials should become increasingly positive (Fig. 14c), in contrast to the experiment by (Mäki-Marttunen et al., 2019a) where the effect was negative. The interested reader can download the code of our model and simulations are available on the Open Science Framework (https://osf.io/ng65r) to generate these and other predictions or to fit our model to their own data sets by following the instructions in the readme file.

The model introduced above is a computational-level theory. It defines the function of meta-control over proactivity. As proposed by Lieder et al. (2018) and confirmed by Bustamante et al. (2021), we postulate that the meta-control system *learns* to approximate the optimal solution proposed by our model. Applied to proactivity in the AX-CPT, the Learned Value of Control (LVOC) model predicts that if we were to create different versions of the AX-CPT task that require more versus less proactivity, then we should see people’s proactivity gradually increase versus decrease over time.

In addition to testing these predictions, future work will extend our investigation of proactivity from proactive control in the AX-CPT task to proactivity in the real world. This will include investigating whether our formal mathematical measure of goal-directedness (see Section 4 of the Supplementary Material) based on people’s performance in simple laboratory paradigms is predictive of proactivity in the real world. In a parallel line of work, we will extend our model by the aspects of proactivity that are currently missing from it. Referring back to the definitions of proactivity that we started from (Crant, 2000; Parker et al., 2010; Seibert et al., 1999; Siebert & Kunz, 2016), we can see that our model captures that proactivity includes the self-initiated and future-oriented setting of intentions and the active pursuit of those intentions over time. However, the important cognitive processes of deriving long-term goals from personal values, planning, motivation, self-improvement, progress monitoring, self-regulation, and reflection are still missing from our model. Incorporating these additional mechanisms will likely allow our model to capture even higher levels of proactivity that people are likely to exhibit in more naturalistic scenarios. As an intermediate step, we will model goal setting and extend our model of proactivity to other experimental paradigms that have been used to study proactive control, including working memory paradigms (Braver, 2012; Burgess et al., 2011).

Furthermore, future work will supplement our model from a computational-level theory of the function of proactivity with mechanistic models of the underlying meta-decision-making processes and investigate how those processes are shaped by learning. Understanding how proactivity is shaped by learning will be an important step towards developing training interventions for helping people become more proactive.

### Relevance to cognitive neuroscience

Proactive control is an important topic in cognitive neuroscience and investigating its neurocomputational mechanisms will further our understanding of the function of prefrontal cortex and the neuromodulatory systems underlying cognitive control (Braver, 2012; Mäki-Marttunen et al., 2019b). We anticipate that our model will become an important asset in the search for the neural mechanisms of proactivity, akin to how the expected value of control model (Shenhav et al., 2013) has helped us elucidate the neural underpinnings of cognitive control and mental effort (Shenhav et al., 2017).

While previous research has demonstrated that the dorsolateral prefrontal cortex plays an important role in the implementation of proactive control (Mäki-Marttunen et al., 2019b), the neural basis of meta-control over proactivity is less well understood. Based on previous work, we postulate that 1) the cost-benefit analyses that govern the meta-control over proactive control are implemented in the dorsal anterior cingulate cortex (dACC, Shenhav et al., 2013; Shenhav et al., 2017), and 2) that the meta-control decisions made by the dACC are then implemented by the dorsolateral prefrontal cortex (dlPFC, Badre, 2008; Mäki-Marttunen et al., 2019b). Future work might employ neuroimaging methods and pupillometry to test our models and our hypothesis about its neural substrates in the three following ways. First, future work might use fMRI to evaluate how well the event-related activity of the dACC is predicted by the cost and benefit terms postulated by our model. Second, fMRI or fNIRS could be used to investigate how well the control signals selected by our model predict the task-dependent activation of the dlPFC (Mäki-Marttunen et al., 2019b) and the locus coeruleus (Mäki-Marttunen et al., 2019b). Pupillometry could be used to test our model’s predictions about the working memory load and mental effort entailed by the control signals and strategies chosen by our model (Kahneman & Beatty, 1966). Third, EEG or MEG could be used to test our models’ assumptions about the number and nature of meta-control decisions are involved in people’s responses on AY trials. This would allow for a more direct comparison of our basic meta-control model (Fig. 2) against the extended meta-control model (Fig. 6a) and simpler meta-control models (Fig. 6c). If the neural correlates of these meta-control decisions can be identified then future work might also leverage them to test our model’s predictions about how those three stages are affected by the statistical structure of the task, the incentives, and cognitive load.

### Conclusions

We believe that our computational level theory of variation in pro- and reactive control is an important step towards a formal theory of proactivity. We hope that by connecting the concept of proactivity from the management and personal development literatures to laboratory paradigms and computational models of meta-control, our article will help to make the computational challenges of living a good live amenable to rigorous scientific investigation in the laboratory.

## Supplementary Information

ESM 1 (DOCX 388 kb)

## Footnotes

1 This learning process can be formally modeled as Bayesian inference on the probabilities *θ*_*j*, *k*_ of the transitions from state *s*_*j*_ (e.g., stimulus A) to state *s*_*k*_ (e.g., stimulus X). This inference is drawn from all previously observed state transitions (*E*_1, ⋯, *t* − 1_) according to Bayes theorem (i.e., $P\left(\theta |E\right)=\frac{P\left(E|\theta \right)\cdotp P\left(\theta \right)}{P(E)}$). The likelihood function can be modeled in terms of one multinomial distribution for each previous state (i.e., *P*(*S*_*t* + 1_| *S*_*t*_ = *s*_*i*_) = Multinomial(*θ*_*i*, 1_, ⋯, *θ*_*i*, *n*_)) and the prior distribution can be modelled as a Dirichlet distribution (i.e., *P*(*θ*_*i*_) = Dirichlet(1, ⋯, 1)). In this way, the learning process can be modelled according to the analytic update equations of the Dirichlet-multinomial model.
