# Supplementary material for: Toward a formal theory of proactivity
Source: Cogn Affect Behav Neurosci. 2021 Mar 15;21(3):490–508. doi: 10.3758/s13415-021-00884-y (PMC8208939; doi:10.3758/s13415-021-00884-y)
Supplement: Supplementary file 1 — (DOCX 388 kb) [file 13415_2021_884_MOESM1_ESM.docx]

**Supplementary Material**

# Model

We derived our model from the principle that cognitive effort should be allocated according to a rational cost-benefit analysis (Shenhav, et al., 2017). This cost benefit analysis entails calculating expected values across all possible combinations of stimuli. Since the (conditional) probabilities of those stimuli are fractions, the resulting sums are rational numbers, rather than integers. While those resulting coefficients may seem arbitrary, they follow directly from the probabilistic structure of the task and were neither fit to data or hand-picked.

## Rational cost-benefit analysis for proactive intention setting

Considering the traditional AX-CPT, where there is only one A-cue (load = 1) and assuming that $u_{+}=u_{-}=\alpha$, the cue-specific expected benefits of setting an intention in the cue phase are for A and B cues are, respectively

$$\mathbb{E}\left[ \mathrm{Benefit}\left( I=1 \right) | A;\alpha,u_{\Delta t},\mathrm{load}=1 \right]=P\left( \mathrm{AX} | A \right)\cdot\left( \mathbb{E}\left[ R_{set intention}|AX;load=1 \right]\mathbb{-E}\left[ R_{\mathrm{habit}}|\mathrm{AX} \right] \right)+P\left( \mathrm{AY} | B \right)\cdot\left( \mathbb{E}\left[ R_{set intention}|AY; load=1 \right]\mathbb{-E}\left[ R_{\mathrm{habit}}|\mathrm{AY} \right] \right)\cdot u_{+}+u_{\Delta t}=-0.105\cdot\alpha+u_{\Delta t}, (1)$$

and

$$\mathbb{E}\left[ \mathrm{Benefit}\left( I=1,\alpha,u_{\Delta t} \right) | B;\alpha,u_{\Delta t},\mathrm{load}=1 \right]=P\left( BX|B \right)\cdot\left( \mathbb{E}\left[ R_{set intention}|BX;load=1 \right]\mathbb{-E}\left[ R_{\mathrm{habit}}|\mathrm{BX} \right] \right)+P\left( \mathrm{BY} | B \right)\cdot\left( \mathbb{E}\left[ R_{set intention}|BY;load=1 \right]\mathbb{-E}\left[ R_{\mathrm{habit}}|\mathrm{BY} \right] \right)\cdot\alpha+u_{\Delta t}=0.420\cdot\alpha+u_{\Delta t}, (2)$$

where $R_{\mathrm{proactive}}$ and $R_{\mathrm{habit}}$ are the rewards for each cue that would be obtained by proactive control and habitual responding respectively, $u_{\Delta t}$ is the utility of the increase response speed achieved by proactive control, $\alpha=u_{+}=u_{-}$ is the reward for a correct response, and $load$ is the contextual load (number of possible A-cues). For simplicity, we model the cost of setting an intention ($\mathrm{cost}(c_{set intention})$) $\mathrm{by}$the constant $\gamma$ which measures additional costs of proactive control due to forming, memorizing, and implementing the intention.

Following this analysis, we find that for A cues the probability of setting an intention is

$$P\left( I=1 | A; \theta,\mathrm{load} \right)=\frac{\exp\left( -0.105\cdot\alpha+u_{\Delta t}-\gamma\cdot\mathrm{load} \right)}{1+\exp\left( -0.105\cdot\alpha+u_{\Delta t}-\gamma\cdot\mathrm{load} \right)}, (3)$$

whereas for B cues the probability of setting an intention is

$$P\left( I=1 | B; \theta,\mathrm{load} \right)=\frac{\exp\left( 0.420\cdot\alpha+u_{\Delta t}-\gamma\cdot\mathrm{load} \right)}{1+\exp\left( 0.420\cdot\alpha+u_{\Delta t}-\gamma\cdot\mathrm{load} \right)}. (4)$$

Since intention setting either occurs or doesn’t occur, the probability of not setting an intention ($I=0)$ is is $P\left( I=0 | cue;\alpha,u_{\Delta t},\gamma\right)=1-P\left( I=1 | cue; \alpha,u_{\Delta t},\gamma\right).$

## Rational cost-benefit analysis of inhibiting/boosting the intention

The expected benefits of exerting the control signal $c$ for X and Y assuming $u_{+}=u_{-}=\alpha$ are given by

$$\mathbb{E}\left[ \mathrm{Benefit}\left( c \right)| X; \alpha,u_{\Delta t},\mathrm{load} \right] =\left( \left( P\left( BX|X \right)\cdot\left( \mathbb{E}\left[ R_{set intention}|BX;load \right]\mathbb{-E}\left[ R_{\mathrm{habit}}|\mathrm{BX} \right] \right)+P\left( \mathrm{AX} | X \right)\cdot\left( \mathbb{E}\left[ R_{set intention}|AX;load \right]\mathbb{-E}\left[ R_{\mathrm{habit}}|\mathrm{AX} \right] \right) \right)\cdot\alpha_{AX}+u_{\Delta t} \right)\cdot c=\left( 0.1055\cdot\alpha_{AX}+u_{\Delta t} \right)\cdot c, (5)$$

and

$$\mathbb{E}\left[ \mathrm{Benefit}\left( c \right)| Y; \alpha,u_{\Delta t}, \mathrm{load} \right] =\left( \left( P\left( BY|Y \right)\cdot\left( \mathbb{E}\left[ R_{\mathrm{proactive}}|AY;load \right]\mathbb{-E}\left[ R_{\mathrm{habit}}|\mathrm{AY} \right] \right)+P\left( \mathrm{BY} | Y \right)\cdot\left( \mathbb{E}\left[ R_{\mathrm{proactive}}|BY;load \right]\mathbb{-E}\left[ R_{\mathrm{habit}}|\mathrm{BY} \right] \right) \right)\cdot\alpha+u_{\Delta t} \right)\cdot c=\left( -0.4200\cdot\alpha+u_{\Delta t} \right)\cdot c, (6)$$

respectively.

## Modeling meta-control over recalling the rules

### We assume that, when engaged, the process of recalling the cue and applying the rules will yield the correct response with a probability of $1-\gamma\cdot\left( \mathrm{load}-1 \right)$, where $\mathrm{load}$ is the number of possible A-cues (contextual load). When no control is exerted, we assume habitual responding to the probe according to Herrnstein’s matching law (Herrnstein, 1961). We therefore assume that for $u_{+}=u_{-}=\alpha$ the benefits of engaging in controlled processing for X and Y probes are given by, respectively

$$\mathbb{E}\left[ \mathrm{Benefit}\left( c_{\mathrm{recall}}=1 \right)|X; \alpha,u_{\Delta t}, \mathrm{load} \right]=P\left( \mathrm{AX} | X \right)\cdot\left( \mathbb{E}\left[ R_{recall rules}|AX;load \right]\mathbb{-E}\left[ R_{\mathrm{habit}}|\mathrm{AX},\alpha\right] \right)+P\left( \mathrm{BX} | X \right)\cdot\left( \mathbb{E}\left[ R_{recall rules}|BX;load \right]\mathbb{-E}\left[ R_{\mathrm{habit}}|\mathrm{BX} \right] \right)\cdot\alpha-u_{\Delta t}=0.2188\cdot\alpha-u_{\Delta t}, (7)$$

and

$$\mathbb{E}\left[ \mathrm{Benefit}\left( c_{\mathrm{recall}}=1 \right)|Y; \alpha,u_{\Delta t}, \mathrm{load} \right]=P\left( \mathrm{AY} | Y \right)\cdot\left( \mathbb{E}\left[ R_{recall rules}|AY;load \right]\mathbb{-E}\left[ R_{\mathrm{habit}}|\mathrm{AY} \right] \right)+P\left( \mathrm{BY} | Y \right)\cdot\left( \mathbb{E}\left[ R_{recall rules}|BY;load \right]\mathbb{-E}\left[ R_{\mathrm{habit}}|\mathrm{BY} \right] \right)\cdot\alpha-u_{\Delta t}=0.1250\cdot\alpha-u_{\Delta t}. (8)$$

## Modeling meta-control over proactive and reactive control in an AX-CPT with no-go trials

When the experiment includes no-go trials on which correctly withholding the response yields a reward of $\alpha$ more units of reward than responding, then the expected benefit of intention setting in response to an A-cue is given by

$$\mathbb{E}\left[ \mathrm{Benefit}\left( c_{set intention}=1 \right) | A;\alpha,u_{\Delta t},load \right]=P\left( \mathrm{AX} | A \right)\cdot\left( \mathbb{E}\left[ R_{\mathrm{proactive}}|AX;load \right]\mathbb{-E}\left[ R_{\mathrm{habit}}|\mathrm{AX} \right]+u_{\Delta t} \right)+P\left( \mathrm{AY} | B \right)\cdot\left( \mathbb{E}\left[ R_{\mathrm{proactive}}|AY;load \right]\mathbb{-E}\left[ R_{\mathrm{habit}}|\mathrm{AY} \right]\cdot\alpha+u_{\Delta t} \right)-P\left( AN | A \right)\cdot\alpha, (9)$$

where $N$ is the probe that signals that the current trial is a no-go trial. Likewise, given a B-cue, the expected benefit of exerting control becomes

$$\mathbb{E}\left[ \mathrm{Benefit}\left( c_{set intention}=1 \right) | B;\alpha,u_{\Delta t},\mathrm{load} \right]=P\left( \mathrm{BX} | B \right)\cdot\left( \mathbb{E}\left[ R_{\mathrm{proactive}}|BX;load \right]\mathbb{-E}\left[ R_{\mathrm{habit}}|\mathrm{BX} \right]+u_{\Delta t} \right)+P\left( \mathrm{BY} | B \right)\cdot\left( \mathbb{E}\left[ R_{\mathrm{proactive}}|BY;load \right]\mathbb{-E}\left[ R_{\mathrm{habit}}|\mathrm{BY} \right]\cdot\alpha+u_{\Delta t} \right)-P\left( BN | A \right)\cdot\alpha. (10)$$

When the N-probe occurs after an intention has been set, then the benefit of exerting control to inhibit the intention is given by

$$\mathbb{E}\left[ \mathrm{Benefit}\left( c \right)| N; \alpha\right]=P\left( inhibit intention | c \right)\cdot\alpha=\left( 1-\left( 0.7+c \right) \right)\cdot\alpha=\left( 0.3-c \right)\cdot\alpha. (11)$$

The cost of doing so is $\mathrm{cost}\left( c \right)=\exp\left( \delta\cdot\left| c \right| \right)-1$. Hence, the net benefit of inhibiting an intention on no-go trials is $\left( 0.3-c \right)\cdot\alpha-\exp\left( \delta\cdot\left| c \right| \right)+1.$ The optimal control signal intensity is $c^{\star}=-log(\alpha)$ assuming that $\alpha>1$, which we deem plausible when participants receive auditory feedback about their performance.

When the N-probe occurs and no intentions was set previously, then the expected benefit of engaging control is $-u_{\Delta t}$. The cost of doing so is $\gamma$. Hence, the net benefit of recalling the rules on a no-go trial when no intention was previously set is $-u_{\Delta t}-\gamma.$ Therefore, the model predicts that people should never recall intentions when they encounter the no-go probe and have not formed an intention yet.

1. **Model fitting**

We used the maximum likelihood estimation method described in Section 2.1 to estimate the parameters $\theta$ of our meta-control process models (see Table 2) and the parameters of our measurement model ($p_{\mathrm{intention}}$ and $p_{\mathrm{recall}}$ and $\lambda$) to the accuracies of individual participations for all datasets shown in Table 2.

Table 1

*Description of the model’s free parameters.*

| Free Parameters | Theoretical Meaning |
| --- | --- |
| $u_{-}$ | Reward for accurate responses for AY, BX and BY trials. |
| $u_{+}$ | Reward for accurate responses for AX trials. |
| $u_{\Delta t}$ | Reward for making an accurately fast response. |
| $\lambda$ | Cognitive load interference. |

- 1. **Maximum likelihood estimation**

We estimated the model parameters $\theta$ from the participants’ accuracies in the AX-CPT using Maximum Likelihood Estimation (MLE). To do so we modelled the number of accurate choices ($N_{cue,probe}^{\left( \mathrm{correct} \right)}$) on a given trial type that occurred $n_{cue,probe}$ times by the Binomial distribution

$$P\left( N_{cue,probe}^{\left( \mathrm{correct} \right)}=k | \mu_{cue,probe} \right)=\binom{n_{cue,probe}}{k}\cdot\left( \mu_{cue,probe}\left( \theta\right) \right)^{k}\cdot\left( 1-\mu_{cue,probe}\left( \theta\right) \right)^{n_{cue,probe}-k}, (12)$$

where $\mu_{cue,probe}(\theta)$ is the accuracy that our model predicts for this trial type when its parameters are set to $\theta$. The likelihood of all of a participant’s choices is then given by the product of the likelihoods of his or her numbers of correct responses across all trial types, that is $P\left( D|\theta\right)=P\left( n_{\mathrm{AX}}^{\left( \mathrm{correct} \right)}| \mu_{\mathrm{AX}}(\theta) \right)\cdot P\left( n_{\mathrm{AY}}^{\left( \mathrm{correct} \right)}| \mu_{\mathrm{AY}}(\theta) \right)\cdot P\left( n_{\mathrm{BX}}^{\left( \mathrm{correct} \right)}| \mu_{\mathrm{BX}}(\theta) \right)\cdot P\left( n_{\mathrm{BY}}^{\left( \mathrm{correct} \right)}| \mu_{\mathrm{BY}}(\theta) \right)$. The likelihood of a dataset is the product of the likelihoods across all participants. Alternatively, under a fixed-effects assumption, all participants’ responses for a given trial type can be treated as if they had been generated by the same participant. This likelihood model allowed us to estimate the model parameters $\theta$ from the data set $D$ of a participant’s choices by maximizing the likelihood function $P\left( D|\theta\right)$ using the standard constrained optimization function fmincon from Matlab’s Optimization Toolbox. The code is available on the Open Science Framework (<https://osf.io/ng65r>).

To fit the parameters $\theta=(u_{+},u_{-},u_{\Delta t},\lambda)$ of the meta-control process model defined in Equations 1-6 of the Main Text, we apply the maximum likelihood estimation method described in the previous paragraph with $\mu_{\mathrm{cue},\mathrm{probe}}\left( \theta\right)=P_{\mathrm{model}}(R=1|cue,probe,\theta)$ as defined in Equation 6 of the Main Text and $\gamma=\frac{1}{3}$. To estimate the parameters $p_{\mathrm{intention}}$, and $p_{\mathrm{recall}}$ of the measurement model defined in Equation 7 of the Main Text, we apply the maximum likelihood estimation method described in the first paragraph with $\mu_{\mathrm{cue},\mathrm{probe}}\left( \theta\right)=P_{\mathrm{measurement}}(R=1|cue,probe,\theta)$ as defined in Equation 7 of the Main Text. To estimate separate parameters for A-trials versus B-trials, we apply the method described above separately to the subsets of A-trials and the subset of B-trials. The same approach can also be used to obtain separate parameter estimates for X-trials versus Y-trials.

Table 2

*Relative trial type frequency and design of the datasets used to fit the model.*

| Study | AX | AY | BX | BY | AN | BN | Other design features |
| --- | --- | --- | --- | --- | --- | --- | --- |
| Mäki-Marttunen (2019) | .70 | .10 | .10 | .10 | - | - | Contextual load and reward manipulation |
| Gonthier (2016)  Experiment 1 | .40 | .10 | .10 | .40 | - | - | Baseline and training strategy |
| Gonthier (2016)  Experiment 2 | .32 | .08 | .08 | .32 | .10 | .10 | Baseline and no-go trials |
| Redick (2014)  AX-CPT-10 | .10 | .70 | .10 | .10 | - | - | Participants with low/high working memory scores |
| Redick (2014)  AX-CPT-40 | .40 | .40 | .10 | .10 | - | - |  |
| Redick (2014)  AX-CPT-70 | .70 | .10 | .10 | .10 | - | - |  |

# Supplementary Results

Table 3

*Mean, standard deviation and independent t-tests for predicted accuracies across individual differences in working memory capacity for AX trials in the experiment by Redick (2014).*

| AX-ACPT version | AX accuracy  low-WM | | AX accuracy  high-WM | | *t* | *df* | *p* |
| --- | --- | --- | --- | --- | --- | --- | --- |
|  | *Mean* | *SD* | *Mean* | *SD* |  |  |  |
| AX-CPT-70 | .9272 | .039 | .9585 | .018 | 3.2472 | 38 | .0024 |
| AX-CPT-40 | .8703 | .086 | .9354 | .038 | 3.0899 | 38 | .0037 |
| AX-CPT-10 | .7127 | .138 | .8627 | .098 | 3.9672 | 38 | .0003 |

- 1. **Fitting the meta control model to the experiments reported by Redick (2014)**

We modeled individual differences in working memory capacity by increasing the cognitive load for participants with low working memory scores from load = 1 to load = 2. Figure 1 compares the accuracies predicted by the fitted model to the empirical data. As summarized in Table 3 and Figure 1, the model was able to capture that participants with low working memory capacity (low-WM) are less accurate in AX trials in all AX-CPT versions.

| **Redick (2014)**  **AX-CPT-70**  ****  **** | **Redick (2014)**  **AX-CPT-40**  ****  **** | **Redick (2014)**  **AX-CPT-10**  ****  **** |
| --- | --- | --- |

*Figure 1*. Mean predicted (upper) and observed (lower) accuracies for Redick (2014) datasets.

- 1. **Fitting the meta control model to the experiments reported in Gonthier et al. (2016)**

The experiments by Gonthier et al. (2016) used within-subjects designs. In Experiment 1, Gonthier et al. (2016) compared participants’ accuracies between the traditional AX-CPT with controlling for the frequency of A and B trials with an experimental condition in which participants were taught to use a proactive strategy. The taught strategy asked participants to prepare for a response during the inter-stimulus interval if they saw an A-cue. This strategy training should increase the participant’s probability to set an intention and thereby decrease their response times in AX trials. In order to maintain their performance on AY trials, participants would have to more frequently inhibit the intention they set in response to the A cue. Therefore, we expect a higher utility to respond faster for the training condition. As show in the left panel of Figure 2, our model was able to capture the decrease in accuracy for AY trials for training condition. Paired two-sample t-tests showed that the utility of responding faster, $u_{\Delta t}$, was higher for the training condition (*M* = .66, *SD* = .70) than for the baseline condition (*M* = .0.35, *SD* = .42), *t*(75) = 4.0684, *p* = .00012). There were no significant changes across conditions in fitted reward parameters, $u_{+}$ and $u_{-}$ (*p* > .14), and interference of cognitive load, $\lambda$ (*p* = .68).

Experiment 2 compared the traditional AX-CPT with a version with the addition of no-go trials. In these no-go trials (AN and BN trials) the cue is followed by a number and participants have to withhold their response. According to our model, the addition of no-go trials should decrease the benefit of setting an intention for both A trials and B trials. In the main text we showed that our model can capture this effect in terms of how the possibility of no-go trials changes the costs and benefits of proactive control. As an additional test of whether the DMC model is sufficient to capture the effect of this manipulation, we fitted an extended version of our model with separate sets of parameters for the baseline condition and the condition with no-go trials to data from both conditions independently. As shown in the right panel of Figure 2, our model was able to capture the decrease in accuracy for BN trials when compared to AN trials, although the absolute predicted accuracy was higher than the observed one.

| **Gonthier et al. (2016)**  **Experiment 1**  **** | **Gonthier et al. (2016)**  **Experiment 2**  **** |
| --- | --- |

*Figure 2.* Mean fitted and observed accuracies for Gonthier et al. (2016) experiment 1 and experiment 2 datasets.

# Quantifying goal-directedness and reactivity

Previous work has developed task-specific behavioral measures of proactive control in the AX-CPT (Cooper, Gonthier, Barch, & Braver, 2017) and self-report measures of proactivity in the real-world (Belwalkar, & Tobacyk, 2018). Existing behavioral measures of proactivity are specific to and limited to very simple tasks, such as the AX-CPT, that do not capture the complexity of proactivity in the real world and do not always involve personally valued goals. Thus, there is currently no general objective, behavioral measure of proactivity that can be applied across various experimental, naturalistic, and simulated settings. Furthermore, while previous measures were designed to differentiate proactive control from reactive control, our measure was designed to quantify their combined effect on goal-directedness, a fundamental characteristic of proactivity. We introduce two equations that can be used to calculate how proactively and how reactively an agent behaved from the relative frequencies with which they emitted different responses across different situations. These metrics were developed independently of the meta-control model of proactivity. They are similar to previous metrics of proactivity and reactivity in the AX-CPT task but they are more general in that they can be applied to arbitrary tasks and they were derived from first principles. Our measure takes into account that reactive control can make a positive contribution to goal-directedness that is complementary to the contribution of proactive control (Mäki-Marttunen, Hagen, & Espeseth, 2019). We demonstrate the validity of our measure by applying it to simulated data from a reinforcement learning task and human data from the AX-CPT.

## 4.1. Formal measures of goal-directedness and reactivity

What do we need to know to predict a person’s actions ($A$)? If the person is completely reactive, then their behavior can be very well predicted from the stimuli $S$ that they are reacting to. In this case, the stimuli provide a large amount of information about the person’s behavior. As the person becomes more proactive, the stimuli around them become increasingly less informative about what they are going to do. Instead, to predict the behavior of a proactive person, we need to know what her goals and values are. Therefore, as a person becomes more proactive, her goals become increasingly more informative about her behavior.

Loosely inspired by Koechlin and Summerfield (2007), we quantify the informativeness of stimuli and goals about a person’s behavior using concepts from information theory (MacKay, 2003). To do so, we first introduce some basic terms from probability theory and information theory. Since we can never be sure about what a person will do, our belief about which action they will take is best described by a probability distribution $P(A)$ that expresses our degree of belief $P(A=a)$ that the person will take action $a$. The less likely the person’s action $a$ is according to our belief $P(A=a)$ the more surprised we will be. Congruent with this intuition, the surprise about this event is formally defined as $-\log P(A=a)$. Not knowing which action $a$ will be taken, the amount of surprise that we can expect on average is $\mathbb{E}_{P(A)}\left[ -\log P(A) \right]=\sum_{i} P\left( A=a_{i} \right)\cdot\left( -\log P\left( A=a_{i} \right) \right).$

Here, we propose to measure goal-directedness by how much information we gain about a person’s behavior as we learn about her goal(s) $\boldsymbol{g}$. Formally, updating our beliefs about a person’s actions based on knowing their goals corresponds to switching from the prior distribution $P(A)$ to the posterior distribution $P(A|\boldsymbol{g})$. The more information we have about what a person is likely to do, the less surprised we will be by their behavior on average. The amount of information we gain about the person’s action $A$ can thus be measured by how much less surprising the agent’s actions are given its goals $\boldsymbol{g}$ compared to how surprising they would be if the agent did not have any goals. This notion can be formalized by the Kullback-Leibler divergence between those two probability distributions. Formally, the Kullback-Leibler divergence from the probability distribution $q$ to the probability distribution $p$ is defined as $\mathrm{KL}\left[ P \right|\left| Q \right]=\mathbb{E}_{P\left( x \right)}\left[ -\log Q\left( x \right) \right]-\mathbb{E}_{P\left( x \right)}\left[ -\log P\left( x \right) \right]$. Based on this concept, we can formally define a person’s goal-directedness from time $t$ to time $T$ as the information that we gain about their actions $A_{t},\cdots,A_{T}$ and the resulting situations and stimuli $S_{t},\cdots,S_{T}$ by learning about their goals beyond what we would be able to predict based on their beliefs $B_{t}$ alone, that is

$$\mathrm{GD}_{\left[ t,T \right]}\left( \mathbf{g} \right)=KL\left[ P\left( \left( A_{t},S_{t} \right),\cdots,\left( A_{T},S_{T} \right) | G_{t}=\boldsymbol{g,}B_{t}=b \right) || P\left( \left( A_{t},S_{t} \right),\cdots,\left( A_{T},S_{T} \right) | G_{t}=\boldsymbol{\emptyset,}B_{t}=b \right) \right], (13)$$

where $P\left( \left( A_{t},S_{t} \right),\cdots,\left( A_{T},S_{T} \right) | G_{t}=\boldsymbol{\emptyset,}B_{t}=b \right)$ is the probability that the person would take actions $A_{t},A_{t+1},\cdots,A_{T}$ and experience the situations $S_{t},S_{t+1},\cdots,S_{T}$ if they had started out without any deliberate goals at time $t$, whereas $P\left( \left( A_{t},S_{t} \right),\cdots,\left( A_{T},S_{T} \right) | G_{t}=\boldsymbol{g,}B_{t}=b \right)$ is the equivalent probability distribution for an agent that starts out with the goal system $\boldsymbol{g}$. Consequently, this formal definition captures the traditional characteristics of proactivity such as the self-initiation, the pursuit of long-term goals and motivation. Furthermore, goal-directedness also depends on many additional cognitive and meta-cognitive characteristics that determine the agent’s self-regulation. In the context of the standard AX-CPT, $S$ is the trial type, $A$ denotes the correctness of the participant’s response, and $P(S,A)$ is the product of the trial type’s frequency and the participant’s accuracy on that trial type. The goal $\boldsymbol{g}$ is to perform the AX-CPT correctly. Not having a goal ($G_{t}=\boldsymbol{\emptyset}$) entails probability matching, and the belief state $b$ is not relevant in this simple scenario.

Following the same logic, we can define reactivity by how much information we gain about a person’s actions when we learn about the situations and stimuli ($S_{t},\cdots,S_{T}$) that they will be presented with, that is

$\mathrm{Reactivity}_{\left[ t,T \right]}=KL\left[ {P(A_{t},\cdots,A}_{T}\left| S_{t},\cdots,S_{T}, G_{t}=\boldsymbol{g},B_{t}=\boldsymbol{b} \right)\boldsymbol{||}{P(A}_{t},\cdots,A_{T}\left| G_{t}=\boldsymbol{g},B_{t}=\boldsymbol{b} \right) \right], (14)$

where $P(a_{t},\cdots,a_{T} \left| S_{t},\cdots,S_{T}, G_{t}=\boldsymbol{g},B_{t}=\boldsymbol{b} \right)$ is the probability that a person with the initial goals $\boldsymbol{g}$ and initial beliefs $\boldsymbol{b}$ will take actions $a_{t},\cdots,a_{T}$ when presented with the stimuli $\mathrm{Sti}m_{t,},\cdots,\mathrm{Sti}m_{T}$. The definition of reactivity thereby emphasizes that how reactive a person is depends on the information gained by knowing the stimuli, controlling for their goals and beliefs in both of the action probability distributions. Both metrics can take values from 0 to infinity because they quantify how much the agent’s behavior deviates from what would be expected if the agent’s actions were independent of its goals (proactivity) or the stimuli that it reacts to (reactivity), respectively.

## Face validity on simulated data

In this section we validate our measures of goal-directedness and reactivity by applying them to simulations of Pavlovian behavior versus goal-directed behavior (van der Meer, Kurth-Nelson, & Redish, 2012). We show that our metrics correctly indicate that goal-directed behavior is more proactive than habitual behavior whereas habitual behavior is more reactive than goal-directed behavior. Furthermore, we show that good decision makers are more proactive than less rational decision-makers.

As a proof of concept, we computed the pro- and reactivity of a goal-directed agent versus a Pavlovian agent in a grid world. The goal-directed agent is the prototype of a proactive agent in that its behavior is entirely controlled by its goal and the cost of its actions. The goal-directed agent $G$ probabilistically chooses the actions that minimize its distance to its goal $g$ (e.g. the top right square), that is$P_{G}\left( A=a | s,g \right)=\frac{\exp\left( -\eta\cdot\left( \mathrm{distance}\left( \mathrm{next}\left( s,a \right),g \right) \right) \right)}{\sum_{k} \exp\left( -\eta\cdot\left( \mathrm{distance}\left( \mathrm{next}\left( s,k \right), g \right) \right) \right)},$ where $next(s,a)$ is the location that the goal-directed agent expects to get to by taking action $a$ in location $s$, the $\mathrm{distance}(s_{1},s_{2})$ is the Manhattan distance from location $s_{1}$ to location $s_{2}$, and $\eta$ is the inverse decision temperature, which determines how much more likely the agent is to choose agents that are more effective at reducing the distance to its goal over actions that are less effective at reducing this distance. For simplicity, we assume that, in the absence of any goal, the goal-based agent falls back on its impulses and acts like the Pavlovian agent described in the following paragraph.

By contrast, the Pavlovian agent is the prototype of a reactive agent. It chooses its actions in reaction to what is immediately in front of it. It is attracted to immediate rewards ($r$) and chooses its actions accordingly, that is$P_{\mathrm{Pavlovian}}\left( A=a | s,g \right)=\frac{\exp\left( \eta\cdot r\left( s,a,\mathrm{next}\left( s,a \right) \right) \right)}{\sum_{k} \exp\left( \eta\cdot r\left( s,a,\mathrm{next}\left( s,k \right) \right) \right)},$ where $\eta$ is the inverse decision temperature which determines to which extent the Pavlovian agent prefers actions with higher immediate rewards over actions with lower immediate rewards. The rewards of the Pavlovian agent were 0 for idleness, -1 for moving left or right, and -3 for moving up or down. We chose these rewards to capture that in most states there are no immediate rewards dangling right in front of the agent, acting is costlier than being idle, and some types of actions are costlier than others. As shown in Figure 3, our formal measure of goal-directedness correctly identifies that the Pavlovian agent is not goal-directed at all and that the goal-based agent becomes more goal-directed as it transitions from choosing randomly to minimizing the distance to its goal as its inverse decision-temperature $\eta$ increases from 0.1 to 10. Furthermore, it also correctly identifies that the reactivity of the Pavlovian agent increases with its inverse decision temperature ($\eta$), whereas the reactivity of the goal-directed agent is zero regardless of its inverse decision temperature. We have thereby validated our formal measures of goal-directedness and reactivity on simulated data. Next, we validate these measures on empirical data from one of the most widely used paradigms for studying proactive control: the AX continuous performance task (AX-CPT).

*Figure 3*. Measuring goal-directedness and reactivity of goal directed and Pavlovian agents.

## Face validity on empirical data from the AX continuous-performance task

The defining feature of proactive control is the initiation of controlled processing in anticipation of future events (Braver, 2012). The AX-continuous performance task was developed to investigate this phenomenon. In this task, participants are presented with a continuous stream of letters that can be grouped into pairs that each comprise a cue (A or B) and a probe (X or Y). The participant’s task is to detect the occurrence of the pair AX by pressing a dedicated key while pressing another key for all other combinations. Critically, in the standard version of the AX-CPT the pair AX occurs 70% of the time whereas the other pairs (AY, BX, and BY) each occur only 10% of the time. Thus, when the participant sees the letter A they can predict that the next letter will most likely be an X and therefore form and memorize the intention to respond affirmatively once the next letter appears. If, despite the odds, the next letter is a Y then they have to recognize the discrepancy and inhibit their prepotent response. This does not always succeed.

To validate our formal measures of goal-directedness and reactivity, we applied them to people’s response frequencies in the AX-CPT experiment conducted by (Mäki-Marttunen et al., 2019). To apply our formal measure of goal-directedness, we used Herrnstein’s matching law (Herrnstein, 1961) to model what people’s response frequencies would have been if they did not have the goal to detect AX pairs. That is, we assume that in the absence of this goal, people would give each response with the frequency that it is rewarded in response to the current stimulus, considering that people learned the rate of occurrence of pairs. Hence, the affirmative response would be attempted 7 out of 8 times that an X is presented and 0 out of 2 times when a Y is presented. Since people do not always succeed in giving the response they intended to give, we assume that there is a 2% chance, based on the error rate on BY trials, that they will accidentally give the opposite response to the one they intended. Similarly, to apply our measure of reactivity, we model what people’s response distributions would be if the current stimulus had no effect on their immediate behavior. In this case, this entails that people should respond affirmatively 7 out of 10 times regardless of which stimulus is presented. We again assumed a 2% chance that their response may be accidentally flipped. We first applied our measures of goal-directedness and reactivity to the response frequencies of all participants in the experiment by (Mäki-Marttunen et al., 2019) individually to obtain one goal-directedness score one reactivity score for each of the 266 participants. Individual participants goal-directedness scores ranged from 0.0846 to 1.0059 (*M* = $0.4316$, *SD* = 0. 1276). We performed one-sample t-tests on those scores to determine whether their population means were significantly larger than zero. Consistent with the fact that this task was designed to investigate the interplay of proactive control and reactive control, we found that people exhibited statistically significant above zero levels of both goal-directedness (*M =* $0.4316$, *SD =* 0. 1276, *t*(265) = 55.1815, *p* < .0001) and reactivity (*M =* $5.1231$, *SD =* 1.9531, *t*(265) = 42.7802, *p* < .0001).

Table 4

*Pearson correlation between the formal measure of goal-directedness and reactivity and traditional measures in the AX-CPT (Braver et al., 2009; Gonthier et al. 2016).*

|  | Reactivity | Goal-Directedness | Accuracy | Mean RT | *d'* context | A-cue bias | PBI-error | PBI-RTs |
| --- | --- | --- | --- | --- | --- | --- | --- | --- |
| Goal-Directedness | 0.24*** |  |  |  |  |  |  |  |
| Accuracy | 0.76*** | 0.12 |  |  |  |  |  |  |
| mean RT | 0.01 | -0.33*** | 0.11 |  |  |  |  |  |
| *d'*-context | 0.53*** | 0.66*** | 0.76*** | -0.14* |  |  |  |  |
| A-cue bias | -0.41*** | 0.68*** | -0.59*** | -0.30*** | 0.00 |  |  |  |
| PBI-errors | 0.04 | 0.70*** | 0.10 | -0.30*** | 0.55*** | 0.50*** |  |  |
| PBI-RTs | 0.01 | 0.04 | 0.03 | 0.14* | 0.05 | 0.03 | 0.09 |  |
| PBI-comp | 0.04 | 0.49*** | 0.09 | -0.10 | 0.39*** | 0.34*** | 0.72*** | 0.76*** |

* indicates *p* < .05. ** indicates *p* < .01. *** indicates *p* < .001.

As summarized in Table 4, we found that our formal measures of proactivity and reactivity exhibited an expected pattern of correlations with related behaviors and extant measures of proactivity and reactivity (i.e., d’-context, A-cue bias, PBI-errors, PBI-RTs, and PBI-comp; Braver et al., 2009; Gonthier et al. 2016)

The extent to which individual participants’ response frequencies differed depending on the currently presented stimulus (reactivity) was highly correlated with their accuracies (*r*(264) = 0.76, *p* < .0001) but not with their reaction times ($r(264)=-0.01, p=.82$). This makes sense given that reacting positively to the letter X and negatively to all other letters regardless of the context was sufficient to achieve an accuracy of 90%. There was a significant negative correlation between goal-directedness and reaction time ($r(264)=-0.33, p<.001$) suggesting that exerting more proactive control allowed participants to respond faster. This makes sense given that proactive control initiates a response to the probe already before it has been presented. As one would expect from the definitions of goal directedness and reactivity for the AX-CPT, there was a statistically significant positive correlation between people’s goal-directedness and reactivity (*r*(264) = 0.246, *p* < .0001) because proactive control and reactive control disagree the response on BX and AY trials but agree on AX and BY trials. These findings further validate our formal measures of goal-directedness and reactivity.

We also used participants’ individual goal-directedness scores to test whether their mean score was higher in the reward condition than in the no-reward condition using a two-sample t-test. As illustrated in Figure 4, we found that participants’ goal-directedness was significantly higher in the reward condition (M = 0.4016, SD = 0.1333) than in the no-reward condition (M = 0.3465, SD = 0.0983; t(264) = 10.4938, p < .0001).

*Figure 4*. On the left, predicted changes in Goal Directedness (KL) for no reward condition (fitted) and the reward condition ($u_{+}+1$ and $u_{\Delta t}+1$). On the right, observed in Goal Directedness (KL) for Mäki-Marttunen et al. (2019a) dataset for load = 1.

## Quantifying the contributions of pro- and reactive control to variations in goal-directedness

Next, we applied our formal measures to quantify how the meta-control processes of intention setting and recalling the rules in response to the probe contribute to goal-directedness and reactivity, respectively. We used the DCM model illustrated in Figure 2 of the main text to simulate the effect of these parameters on behavior and then applied our metrics to the resulting accuracies for each of the four trial types. As illustrated in Figure 5a, we found that varying the parameter $p_{\mathrm{intention}}$ between $0$ and $1$, our model’s behavior spans a continuum ranging from the absence of goal-directedness to a level of goal-directedness that exceeds the average level of goal-directedness observed in people. Interestingly, varying the probability of reactive control ($p_{\mathrm{recall}}$) has a very similar effect. This makes sense because both forms of control increase the effect that the goal of the AX-CPT has on the model’s response probabilities. Reactive control is especially critical for goal-directed behavior when no intention was set, and intention-setting is especially critical when there is no reactive control. Thus, since neither of the two control processes is perfectly reliable, the combination of the two enables a higher level of goal-directedness than either of them could achieve in isolation (see Figure 5a). This indicates that proactive control and reactive control are both essential for goal-directed behavior. Conversely, Figure 5b shows that reactivity scores are predominantly affected by $p_{\mathrm{recall}}$ for high values of $p_{\mathrm{recall}}$, while reactivity is increased by extremes values of $p_{intention}$ when $p_{\mathrm{recall}}$ is low.

| **a)** | **b)** |
| --- | --- |

| 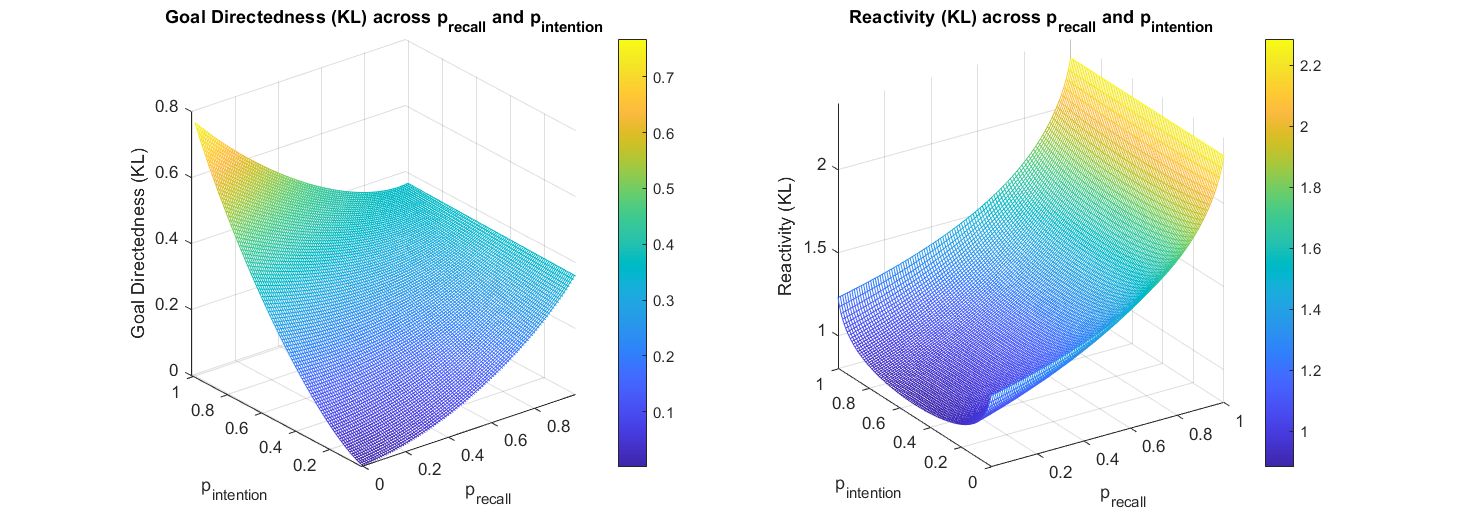 |
| --- |

*Figure 5.* Goal-Directedness and reactivity as a function of the probability to set an intention during the presentation of the cue*,* $p_{\mathrm{intention}}$*,* and the probability to recall the cue and rules*,* $p_{\mathrm{recall}}$*.*

Finally, we investigated how good our meta-control model is at explaining inter-individual differences in goal-directedness and reactivity. We fitted the DCM model (see Figure 2 in the main text) to the data from individual participants in Experiment 1 from Mäki-Marttunen, et al. (2019b) and then compared the predicted accuracies to the participant’s accuracies in terms of our metrics of goal-directedness and reactivity. Concretely, we applied our goal-directedness metric (Equation 13) and our reactivity metric (Equation 14) separately to each participant’s observed accuracies and the corresponding model predictions. We then quantified how well the fitted model was able to capture individual differences in goal-directedness by calculating the correlation between the goal-directedness scores of individual participants to the goal-directedness scores of the corresponding model fits. We found that the levels of goal-directedness and reactivity predicted by the fitted model was highly correlated with the participants’ goal-directedness scores ($r=0.9440$). When we repeated the same analysis for the reactivity scores and found a similar correlation ($r= 0.7261$). Our model thereby explained $89\%$ of the inter-individual variance in goal-directedness and 53% of the inter-individual variance in reactivity. This highlights that our meta-control model is a promising step toward a mechanistic understanding of the effect of situational and inter-individual differences in the extent to which behavior is goal-directed.

**4.5. Relationship to Koechlin and Summerfield’s quantification of executive control**

Although our measures of goal-directedness and reactivity were inspired by Koechlin and Summerfield’s use of information theory to quantify cognitive control, our measures are conceptually and mathematically different. One difference is that our measures explicitly refer to goals whereas Koechlin and Summerfield’s model refers to contextual cues. Leaving this difference aside, our measure of goal-directedness is conceptually similar to the term I(C,A|S) in the account by Koechlin and Summerfield (2007), if we interpret what they term the context C as the agent’s goals **g**. However, one important difference is that we formalize goal-directedness in terms of the control that a person’s goals exert about their actions and their situation whereas the term I(C,A|S) treats the situation as an external factor. This difference allows our measure of goal-directedness to credit the agent for changing its environment and for the effects that those changes have on its future behavior. Our measure of reactivity is conceptually related to Koechlin and Summerfield’s measure of stimulus control (I(S,A)). The main difference is that our measure controls for the effect of the agent’s goals. This makes a difference in scenarios where the stimulus is informative about the agent’s goal. In those scenarios I(S,A) might falsely attribute some of the causal effects of the goal to the stimulus. Our measure avoids this problem by explicitly considering the agent’s goals.

**References**

Belwalkar, B. B., & Tobacyk, J. J. (2018). Toward a tripartite model and measurement of proactive personality. *Journal of Personality Assessment*, *100*(5), 529-538.

Braver, T. S., Paxton, J. L., Locke, H. S., & Barch, D. M. (2009). Flexible neural mechanisms of cognitive control within human prefrontal cortex. *Proceedings of the National Academy of Sciences*, *106*(18), 7351-7356.

Braver, T. S. (2012). The variable nature of cognitive control: a dual mechanisms framework. *Trends in Cognitive Sciences, 16*(2), 106-113.

Cooper, S. R., Gonthier, C., Barch, D. M., & Braver, T. S. (2017). The role of psychometrics in individual differences research in cognition: A case study of the AX-CPT. *Frontiers in Psychology, 8*, 1482.

Gonthier, C., Macnamara, B. N., Chow, M., Conway, A. R., & Braver, T. S. (2016). Inducing proactive control shifts in the AX-CPT. *Frontiers in Psychology*, *7*, 1822.

Herrnstein, R. J. (1961). Relative and absolute strength of response as a function of frequency of reinforcement 1, 2. *Journal of the Experimental Analysis of Behavior, 4*(3), 267-272.

Koechlin, E., & Summerfield, C. (2007). An information theoretical approach to prefrontal executive function. *Trends in Cognitive Sciences, 11*(6), 229-235.

MacKay, D. J. (2003). *Information theory, inference and learning algorithms*: Cambridge university press.

Mäki-Marttunen, V., Hagen, T., & Espeseth, T. (2019). Proactive and reactive modes of cognitive control can operate independently and simultaneously. *Acta Psychologica, 199*, 102891.

Redick, T. S. (2014). Cognitive control in context: Working memory capacity and proactive control. *Acta Psychologica, 145*, 1-9.

van der Meer, M., Kurth-Nelson, Z., & Redish, A. D. (2012). Information processing in decision-making systems. *The Neuroscientist, 18*(4), 342-359.
